# Multistate Density Functional Theory: Theory, Methods, and Applications

**DOI:** 10.1002/wcms.70043

**Published:** 2025-09-18

**Authors:** Yangyi Lu, Jiali Gao

**Affiliations:** 1 Shenzhen Bay Laboratory, Institute of Systems and Physical Biology, Shenzhen, China; 2 College of Chemical Biology and Biotechnology, Beijing University Shenzhen Graduate School, Shenzhen, China; 3 Department of Chemistry and Supercomputing Institute, University of Minnesota, Minneapolis, Minnesota, USA

**Keywords:** Hamiltonian matrix functional, matrix Density, minimal active space, multi-state density functional theory

## Abstract

A quantum theory of density functionals and its applications is presented. By introducing a matrix density D(r) of rank N as the fundamental variable, a one-to-one correspondence has been established between D(r) and the Hamiltonian matrix representing N electronic states—that is, a matrix density functional ℋ[D]. Moreover, no more than $N^{2}$ Slater determinants are sufficient to represent D(r) exactly, giving rise to the concept of minimal active space (MAS). The use of a MAS naturally leads to the definition of correlation matrix functional ${ℰ}^{c}[D]$, the multi-state extension of the exchange-correlation functional in Kohn–Sham DFT. Variational minimization of the multistate energy, which is defined as the trace of the Hamiltonian matrix functional, yields the exact energies and densities of the lowest N eigenstates. A nonorthogonal state interaction (NOSI) algorithm has been developed to optimize the orbitals associated with D(r) and to approximate the correlation matrix functional. The MSDFT-NOSI method is demonstrated across a range of applications, particularly in cases where KS-DFT and linear-response time-dependent DFT fail, with its accuracy validated through comparison with high-level multiconfigurational wave function theory.

## 1 | Introduction

The seminal works of Hohenberg, Kohn, and Sham established the foundation of modern density functional theory (DFT) for the ground state of electronic systems [1, 2]. Since then, tremendous efforts have been made as illustrated by a myriad of applications in physics, chemistry, materials science and biology, which have been featured in a series of reviews in this and other journals [3–16]. The Hohenberg–Kohn theorems assert that the ground-state energy of a system is uniquely determined by its ground-state electronic density, $E_{0}=E\left[\rho_{0}\right]$, which can be variationally optimized if an approximate density is used [1]. Subsequently, Kohn and Sham (KS) proposed that the density functional of the ground state energy can be constructed on the basis of a noninteracting reference system, with the ground state density exactly represented by the Kohn–Sham orbitals [2]. Despite its remarkable success, culminating in the award of the 1998 Nobel Prize in Chemistry to Walter Kohn along with John Pople, the theorems of Hohenberg and Kohn are incomplete as quantum theory. There was no rigorous DFT beyond the ground state other than linear response approaches until recently.

In 2022, the authors proved that the Hamiltonian of a system in the Hilbert subspace spanned by the lowest N eigenstates is a matrix functional of the multistate matrix density D(r), H[D] [17]. In multi-state density functional theory (MSDFT), the fundamental variable is D(r), an N-dimensional matrix of state densities and transition densities. Furthermore, the trace of the Hamiltonian matrix functional (the sum of the diagonal elements) is the variational minimum if D(r) is the exact matrix density of the subspace. This opens up possibilities of developing approximations to the matrix density functional as time-independent approaches in DFT to determine the exact energies and densities of both the ground state and excited states. The aim of this article is to present the development and current status of the quantum theory of density functionals along with its computational methods and applications.

In KS-DFT, the density of the interacting, physical state is represented by a noninteracting reference system, for which an exchange-correlation (xc) functional is introduced to account for the effects of electron correlation [18]. Although the amount of the xc energy only takes up a tiny fraction of the total electronic energy of a given molecular system, it has a major impact on the computational accuracy of chemical properties. Consequently, in the past six decades, heroic efforts have been made in the development of approximate xc functionals, which have seen a remarkable improvement in computational accuracy; KS-DFT has become the method of choice for large systems [19–23]. Meanwhile, the rapidly expanding toolbox of functional approximations extends the applicability of KS-DFT to a wide range of targets [24, 25], from simulation of atoms, molecules [3], and periodic materials [6] to modeling intermolecular properties [26, 27]. Undoubtedly, DFT is currently and in the foreseeable future the most popular electronic-structure method for the general scientific community [5, 18, 28].

For the purpose of accessing excited-state properties, an exact generalization to time-dependent density functional theory (TDDFT) was derived [29, 30]. The Runge–Gross theorem asserts that, given the initial state of a system, the time-dependent properties of the system are uniquely determined by its time-dependent electronic density [31]. Analogous to KS-DFT for the ground state, a KS determinant, consisting of time-dependent orbitals, is introduced to represent the time-dependent electronic density of the physical system. In practice, a linear-response (LR-) formalism of TDDFT is derived by assuming the time-dependent external potential to be a small perturbation such that exact excitation energies of the *time-independent* system can in principle be extracted from a *pseudo-eigenvalue* equation, known as Casida’s equation [32]. Unfortunately, an exchange-correlation kernel that is frequency-dependent is involved in Casida’s equation, whose frequency-dependent behaviors are only marginally understood despite the investment of tremendous efforts. Consequently, most computations in LR-TDDFT are performed under the adiabatic approximation, which neglects the frequency dependence of the xc kernel and relates it to the second functional derivative of the KS xc energy functional. Equipped with the powerful armory of KS functional approximations, LR-TDDFT has been enormously successful in predicting the excitation energies of the class of excited states that are dominated by single-electron excitations [33].

In spite of many successes, LR-TDDFT has failed systematically in studying excited states with distinct characters [34]. These failures can be classified according to their theoretical origins, including double excitation, charge-transfer excitation, conical intersection, and open-shell systems [35–37]. In addition to these failures, LR-TDDFT only computes excitation energies and is incapable of obtaining other relevant physical properties of excited states. This undesirable feature is rooted in the fact that, in LR-TDDFT, excited states are treated as response states in the density response function, which only encodes the information of excitation energies. Moreover, we shall remark that TDDFT has been frequently applied to study real-time dynamics, and additional issues arise in these applications. However, this topic is out of the scope of the current Review. We shall refer interested readers to other excellent reviews [38–41].

The correlation energy of single-reference systems, in which there is a large energy separation between the highest occupied orbital (HOMO) and the lowest unoccupied orbital (LUMO), can be accurately computed by KS-DFT; LR-TDDFT typically works well for excitation energies [5, 30]. However, when the HOMO and LUMO of a system become nearly degenerate, that is, the HOMO-LUMO gap is close to zero, the single-determinant approximation to the ground-state wavefunction breaks down qualitatively, in which case more than one configuration contributes to the ground state, and a multiconfigurational approach is needed.

Multi-reference wavefunction theory (MR-WFT) has the ability to perform a balanced treatment between static and dynamic correlation and therefore has served as the most reliable approach for modeling strongly correlated systems [34, 42–46]. Since static correlation results from near degeneracy of a relatively small number of determinants, effects of static correlation can be accurately accounted for within a small Hilbert subspace (known as the model space). On the other hand, dynamic correlation comes from a large number of high-energy determinants, each of which only makes marginal contributions. Various MR-WFT approaches exist which differ in their choices to address static and dynamic correlation. One of the most popular ways to treat static correlation is the complete-active-space self-consistent-field theory (CASSCF) [47–49]. CASSCF constructs the model space by performing a full-configuration-interaction (FCI) computation within a selected set of orbitals, known as active orbitals, that are relevant to the state(s) of interest. With the multi-determinant wavefunction established in the model space as the reference, dynamic correlation is then included through perturbative expansions using multi-reference perturbation theory (MR-PT), truncated configuration interaction (MRCI), or the coupled-cluster ansatz (MRCC) [42–44, 50, 51].

An excited state can be connected to the ground state through an excitation operator such that the Schrödinger’s equation of the excited state yields an equation of motion (EOM) for the corresponding excitation operator [52]. If both the ground-state wavefunction and the excitation operator are exact, the exact excited-state wavefunction can be obtained. The EOM is solved in practice for a truncated excitation operator acted upon a sufficiently accurate ground-state wavefunction, often computed by the coupled-cluster method; hence, the EOM-CC approach. Therefore, the underlying implicit assumption is that the excited-state wavefunctions must not differ considerably from the reference ground-state wavefunction, which is not always true in molecular systems. Because of the exponential operator ansatz, EOM-CC can capture a fraction of static-correlation effects and therefore produce accurate excited-state properties for weakly to moderately correlated systems [53]. Rowe’s EOM approach has also been applied to compute excited-state properties [54, 55].

A computational scheme that treats all eigenstates of interest on an equal footing is the state average (SA) approach. A frequently used approach is the SA-CASSCF, which performs a state-average computation within the model space constructed by CASSCF. The basic idea of SA is to construct an effective Hamiltonian matrix within a Hilbert subspace, which is followed by a direct diagonalization or Davidson’s method to compute the lowest eigenstates of interest [56]. When active orbitals are properly chosen, the SA-CASSCF approach can be very successful in capturing static correlation in the eigenstates of interest, but sacrificing the true variational ground-state energy [43, 48]. To obtain accurate excitation energies, a direct correction of dynamic correlation to the reference wavefunction from SA-CASSCF by second-order perturbative theory (CASPT2) can be problematic in strongly correlated systems such as conical intersection or avoided crossing. In this case, there can be strong interaction between the wavefunctions of the primary states due to the feedback of dynamic correlation from the secondary space, which can result in qualitative failures. The minimal step in this context is the so-called (restricted) static-dynamic-static approaches developed by Liu and coworkers [57] and to perform the state-average procedure throughout to incorporate all necessary state interactions. This approach is known as the extended multi-state CASPT2 (XMS-CASPT2) [58, 59].

The major advantage of multi-reference WFT approaches is that their performance can be systematically improved, with convergence towards the FCI limit. Sophisticated schemes also exist in MR-WFT that allow flexibility to select relevant configurations for computing eigenstates of interest, without resorting to CAS-based approaches [43, 60, 61]. Nonetheless, accurate MR-WFT approaches typically suffer from the unfavorable scaling in computational cost with system size. This drawback considerably limits the applicability of MR-WFT approaches.

In this Review, we present a comprehensive discussion of MSDFT [17, 62], an exact DFT for all electronic states. In the following, we first introduce the rigorous theoretical foundation of MSDFT and the minimal active space (MAS) concept associated with matrix density representation and correlation matrix functional. Then, we present the non-orthogonal state average (NOSI) algorithm that has been developed for treating both the ground state and excited states of molecular systems. This is followed by a list of applications—particularly emphasizing situations where KS-DFT and LR-TDDFT fail—ranging from excitation energies and strongly correlated systems to charge transfer and excited-state energy transfer reactions. It is hoped that this Review will inspire those interested in extending DFT to broader applications to develop more sophisticated computational methods based on a rigorous theory for all electronic states.

We remark that other classes of computational approaches exist that have an appearance in analogy to MSDFT, but in fact differ fundamentally since they are based on the one-state-DFT framework without explicitly including dynamic correlation through state interaction. Approaches in this category combine multi-reference wave functions with Kohn–Sham density functionals for the purpose of benefiting from both MR-WFT and KS-DFT. To limit the scope of this review, neither do we include work associated with the use of reduced density matrices. We refer interested readers to an excellent review for a comprehensive summary of these methods [63].

## 2 | Background

We consider a system of n electrons (n>1) under the local multiplicative external attractive potential ${\hat{V}}_{\text{ext}}=\sum_{j=1}^{n}v\left(r_{j}\right)$, whose electronic structure is governed by the Hamiltonian ${\hat{H}}_{v}={\hat{H}}_{0}+{\hat{V}}_{\text{ext}}$ with ${\hat{H}}_{0}$ consisting of the kinetic $\left(\hat{T}\right)$ and electronic Coulomb $\left(\hat{W}\right)$ operators,

(1) $${\hat{H}}_{0}=\hat{T}+\hat{W}=-\frac{1}{2}\sum_{j=1}^{n}\nabla_{j}^{2}+\frac{1}{2}\sum_{i\neq j}^{n}\frac{1}{\left|r_{i}-r_{j}\right|}$$

where atomic units are used and $\nabla_{j}^{2}$ is the Laplace operator for the jth electronic coordinate. We denote the exact eigenstates of ${\hat{H}}_{v}$ as $\left|\Psi_{I}\right\rangle ,{\hat{H}}_{v}\left|\Psi_{I}\right\rangle =E_{I}\left|\Psi_{I}\right\rangle$ with the eigenenergies ordered as $E_{1}\leq E_{2}\leq \cdots$. The complete Hilbert space of n-electron square-integrable wavefunctions is denoted by H.

### 2.1 | Prelude

Given a molecular system with the Hamiltonian ${\hat{H}}_{v}$, the ground-state energy $E_{1}$ can be regarded as a scalar functional of the external potential v(r) [1, 64, 65]. Extending this result to excited states, for an arbitrary number of states N≥1, the average energy of the lowest N eigenstates $E_{N}$ is a scalar functional of v(r):

(2) $$E_{N}[v]=N^{-1}\sum_{I=1}^{N}E_{I}$$

In the special case of $N=1,\;E_{1}[\nu ]=E_{1}$, as that described in references [64, 65]. It shall be noted that the above statement is true regardless of whether or not the lowest N eigenstates have degeneracy.

In general, $E_{N}[v]$ can be obtained by variational minimization of the average expectation value of N orthonormal state vectors, not necessarily the eigenstates, over the complete Hilbert space $H,\;\left\{\Phi_{A};A=1,\ldots ,N\right\}\in H$,

(3) $$E_{N}[v]=\underset{\left\{\Phi_{A}\right\}}{min}\;\left\{N^{-1}\sum_{A=1}^{N}\;\left\langle \Phi_{A}\right|\overset{ˆ}{H}\left|\Phi_{A}\right\rangle \right\}$$

where $\left\langle \Phi_{A}|\Phi_{B}\right\rangle =\delta_{AB}$. In fact, the variational principle of Equation (3) is a general property of Hermitian operators [66].

However, there is a critical distinction between a single state (N=1) and multiple states. If the ground state is non-degenerate, the variational principle (3) determines a unique ground-state wavefunction with the ground-state energy $E_{1}[\nu ]$. On the other hand, when N>1, the variational minimization of the average energy does not guarantee a unique set of N states $\left\{\Phi_{A}\right\}$, especially the eigenstates. In fact, the determination of $E_{N}[v]$ broadly results in a unique N-dimensional *Hilbert subspace* $S_{v}^{N}\subset H$ that is spanned by $\left\{\Phi_{A}\right\}$. Importantly, $S_{v}^{N}$ is equivalently spanned by the lowest N eigenstates of the system, $S_{v}^{N}=span\left\{\Psi_{I};I=1,\ldots ,N\right\}$. A special quantity uniquely associated with the subspace $S_{v}^{N}$, just as $E_{N}[v]$, is the average density of all eigenstates, called the *subspace density*, given as follows.

(4) $$\rho_{S}(r)=N^{-1}\sum_{I=1}^{N}\rho_{I}(r)$$

where $\rho_{I}(r)=<\Psi_{I}|\overset{ˆ}{\rho }(r)|\Psi_{I}>$ and $\overset{ˆ}{\rho }(r)=\sum_{j=1}^{n}\delta \left(r-r_{j}\right)$ is the one-electron density operator. It is of interest to point out that neither $E_{N}[v]$ nor $\rho_{S}(r)$ are physical observables.

To determine the N eigenstates, it is necessary to know the Hamiltonian matrix H within $S_{v}^{N}$ spanned by $\left\{\Phi_{A}\right\}$,

(5) $$H_{AB}=\left\langle \Phi_{A}\right|\overset{ˆ}{H}\left|\Phi_{B}\right\rangle$$

for all A,B=1,…,N. Then, diagonalization of H yields the eigenenergies and eigenstate vectors,

(6) $$HC=Cℰ\Rightarrow \Psi_{I}=\sum_{A=1}^{N}C_{AI}\Phi_{A}$$

where $ℰ=diag\left\{E_{1},\ldots ,E_{N}\right\}$ is the N-dimensional diagonal matrix with eigenenergies in the diagonal entries. We shall remark that the subspace $S_{v}^{N}$ is unique if and only if it contains all degenerate eigenstates with the highest energy $E_{N}$. We will make this assumption throughout the rest of this Review.

### 2.2 | Scalar Density Functionals of the Ground State and Subspace

Turning to DFT, we first recall the density functional for the ground state, introduced by Hohenberg and Kohn [1]; and then describe the subspace DFT as an extension [67, 68].

#### 2.2.1 | Ground-State Energy Functional

In the case of a non-degenerate ground state, Hohenberg and Kohn showed that there exists a one-to-one correspondence between the ground-state density and the ground-state energy $E_{1}[v]$ [1]. Following the foundational work, Levy proposed that there exists a universal functional with respect to an arbitrary n-electron density ρ(r), ${\tilde{F}}_{1}[\rho ]$, which can be defined through a constrained minimization of the expectation value of the bare Hamiltonian ${\hat{H}}_{0}$ by searching over all possible wavefunctions Φ∈H that yield the density ρ(r), that is, $\rho (r)=\langle \Phi |\overset{ˆ}{\rho }(r)|\Phi \rangle$ [69],

(7) $${\tilde{F}}_{1}[\rho ]=\underset{\Phi }{min}\;\left\{\langle \Phi |{\overset{ˆ}{H}}_{0}|\Phi \rangle |\langle \Phi |\overset{ˆ}{\rho }|\Phi \rangle =\rho \right\}$$

Lieb later proved the existence of a universal functional through the Legendre–Fenchel transform of the ground-state functional $E_{1}[v]$ [64],

(8) $$F_{1}[\rho ]=\underset{v}{sup}\;\left\{E_{1}[v]-\int drv(r)\rho (r)\right\}$$

where sup means finding the *supremum* of the enclosing expression and ρ(r) and v(r) are called conjugate variables [70].

The two functionals ${\tilde{F}}_{1}[\rho ]$ and $F_{1}[\rho ]$ are closely connected but different in two significant ways. First, ${\tilde{F}}_{1}[\rho ]$ is defined on the domain of n-*representable densities*, denoted as ${𝒲}_{n}$ [64], in which each density must come from a normalized state vector Φ∈H. $F_{1}[\rho ]$ is defined on a greater domain than ${\tilde{F}}_{1}[\rho ]$, enclosing ${𝒲}_{n}$:

(9) $${𝒥}_{n}=\left\{\rho |\rho (r)\geq 0,\rho (r)\in H^{1}\left(R^{3}\right)\right\}$$

where $H^{1}\left(R^{3}\right)$ is the first Sobolev space and ${𝒲}_{n}\subset {𝒥}_{n}$ [71]. Importantly, the inequality always holds,

(10) $$F_{1}\left[\rho \right]\leq {\tilde{F}}_{1}\left[\rho \right],\;\forall \rho \in {𝒲}_{n}$$

Secondly, $F_{1}[\rho ]$ is a convex functional with respect to the variable ρ(r) within the convex domain ${𝒥}_{n}$, but ${\tilde{F}}_{1}[\rho ]$ is not.

Then, the energy functional for the ground state can be defined with respect to the density on the domain ${𝒥}_{n}$ [64].


(11) $$E_{1}[\rho ]=F_{1}[\rho ]+\int drv(r)\rho (r),\;\rho \in {𝒥}_{n}$$

The convexity of $F_{1}[\rho ]$ guarantees that the minimum of $E_{1}[\rho ]$ must exist within ${𝒥}_{n}$ [72, 73]. Therefore, the Hohenberg–Kohn *variation principle* for the ground state can be rigorously stated as

(12) $$E_{1}[v]=\underset{\rho }{min}\;\left\{F_{1}[\rho ]+\int drv(r)\rho (r)|\rho \in {𝒥}_{n}\right\}$$

Kohn and Sham (KS) devised a procedure to use a noninteracting reference system to exactly represent the ground state density, leading to the introduction of an exchange-correlation functional to account for electron correlation of the reference state [2]. Essentially, all ground-state calculations using DFT are based on this scheme known as KS-DFT.

#### 2.2.2 | Subspace Energy Functional

Analogous to Hohenberg–Kohn theory for the ground state, there exists a subspace analog between the scalar subspace density $\rho_{S}(r)$ defined in Equation (4) and the subspace energy $E_{N}[v]$ of $S_{v}^{N}$ in Equation (3) [74]. In contrast to the widespread applications of Hohenberg–Kohn theory, subspace DFT, including the use of non-equal weighting of state densities [75], has seen limited applications, if any, to realistic systems in the literature. Nevertheless, we introduce the universal subspace functional in terms of the Legendre–Fenchel transform [68].

Following the approach of Lieb for the ground-state DFT [64], we apply the Legendre–Fenchel transform to the subspace energy functional (2) of v(r) with the conjugate variable $\rho_{S}(r)$ and obtain the *universal subspace functional* $F_{N}\left[\rho_{S}\right]$,

(13) $$F_{N}\left[\rho_{S}\right]=\underset{v}{sup}\;\left\{E_{N}[v]-\int drv(r)\rho_{S}(r)\right\}$$

Equation (13) may be viewed as a subspace generalization of the Lieb universal functional for the ground state (8).

Similarly, the subspace functional $F_{N}\left[\rho_{S}\right]$ is closed and convex for $\rho_{S}(r)\in {𝒥}_{n}$. Consequently, the subspace variation principle can be expressed as follows:

(14) $$E_{N}[v]=\underset{\rho_{S}}{min}\;\left\{F_{N}\left[\rho_{S}\right]+\int drv(r)\rho_{S}(r)\right\}$$

The similarity between the ground-s tate theory and subspace formulation—a scalar energy functional of a scalar density—has led to attempts to construct an analogous Kohn–Sham scheme for the total energy of a subspace [67, 74]. However, the subspace energy $E_{N}[\nu ]$ and the subspace density $\rho_{S}(r)$ only contain the average information of states. One cannot extract precise information about individual eigenstates solely through the variational principle of Equation (14) or an alternative extension. We shall not be distracted from the main goals of this review by analyzing this issue further. Determination of energies of excited states using the subspace functional requires a sequential, “build-u p” approach that computes subspace energies from one to N dimensions, if this was possible at all.

## 3 | Multi-State Density Functional Theory

In order to directly access the energies and densities of individual eigenstates, it is necessary to establish an entirely new variation principle derived on the basis of a density variable that encompasses state interactions to precisely determine the spectrum of states within the subspace. The multistate theorems of Lu and Gao identify that the N-matrix density D(r) is the fundamental variable for all states within a subspace [17].

### 3.1 | Matrix Density for N Electronic States

For a set of N electronic states, we introduce the N-matrix density, D(r), comprising state densities on the diagonal and transition densities on the off-diagonal. As demonstrated in [76], D(r) is both necessary and sufficient to determine the exact energies of individual eigenstates within the subspace $S_{v}^{N}$. Thus, D(r) naturally serves as the fundamental variable in a DFT encompassing multiple states. Throughout this review, we emphasize that relying solely on a limited set of N state densities is inadequate for pinpointing the properties of each eigenstate. This inadequacy arises because (i) there is no variational principle applicable to individual excited states, and (ii) transition densities are essential to describing state interactions that dictate the eigenstate spectrum.

Extending the concept of density representability for the ground-state [69], we define n-*representable* and v-*representable* N-matrix densities as follows [68]:

- n-*representable*: Given an arbitrary set of N orthonormal state vectors $\left\{\Phi_{A};A=1,\ldots ,N\right\}\in H$, an N-matrix density D(r) is n-*representable* if its elements are determined by

(15) $$D_{AB}(r)=\left\langle \Phi_{A}\right|\overset{ˆ}{\rho }(r)\left|\Phi_{B}\right\rangle$$

In particular, the diagonal elements of the N-matrix density D(r) are the densities of individual states:

(16) $$\rho_{A}(r)\equiv D_{AA}(r)=\left\langle \Phi_{A}\right|\overset{ˆ}{\rho }(r)\left|\Phi_{A}\right\rangle$$

Here, we used two symbols, respectively, with one or two indices, to denote state densities and they are used interchangeably throughout. The off-diagonal elements of D(r) are transition densities (A≠B in Equation 15).

- v-*representable*: An N-matrix density is v-*representable* if D(r) is (i) n-*representable* and (ii) there exists a Hamiltonian ${\hat{H}}_{v}$ such that the N states $\left\{\Phi_{A}\right\}$ for representing D(r) belong to the N-dimensional subspace $S_{v}^{N}\subset H$. Specifically, the subspace $S_{v}^{N}$ is spanned by the corresponding N eigenstates (not necessarily the lowest ones) of the Hamiltonian ${\hat{H}}_{v}$. Therefore, any state $\Phi_{A}\in S_{v}^{N}$ can be written as a linear combination of these eigenstates:

(17) $$\Phi_{A}=\sum_{I}^{N}C_{AI}\Psi_{I}$$

In general, the set of states $\left\{\Phi_{A}\right\}$ can be non-orthogonal as long as they are linearly independent. In this case, the overlap integral between two states will not be zero and can be determined as follows

(18) $$S_{AB}=<\Phi_{A}\left|\;\Phi_{B}>=\frac{1}{n}\int D_{AB}(r)dr\right.$$

These nonorthogonal states can always be transformed into a set of orthogonal states. Thus, the discussion in terms of orthogonal states is equally applicable after the orthogonalization. It is important, however, to note that even when the overlap integral between two states is zero, $D_{AB}(r)$ may not be zero everywhere in space.

**FIGURE 1 |:**
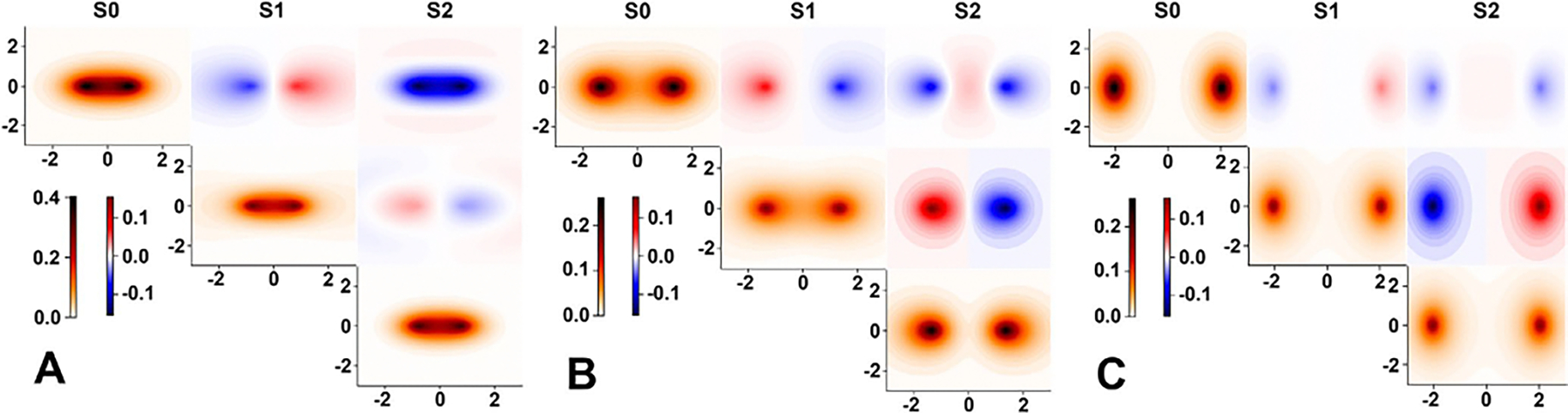
Matrix density of the ground state and the first two singlet excited states of $H_{2}$ at the equilibrium bond distance $R_{eq}=1.40$ bohr (left), at $2R_{eq}$ (middle) and at $4R_{eq}$ (right). Contour levels are shown in the plane cutting through the molecular axis in atomic units (electrons per cubic bohr). Adapted from [76] with permission by the American Chemical Society.

In fact, one property of interest is the transition dipole moment $d_{IJ}$ between states I and J, which can be determined by integrating the corresponding transition density element of $D(r):d_{IJ}=\int r\cdot D_{IJ}(r)dr$. This highlights the critical role of transition densities in determining the spectrum of excited states. We illustrate the matrix density in Figure 1 for the lowest three singlet states of a hydrogen molecule at three different bond lengths. Considering the symmetry properties of transition densities and the spatial coordinates r=(x,y,z), the excitation from the ground state $S_{0}\left(\Sigma_{g}^{+}\right)$ to the first excited state $S_{1}\left(\Sigma_{u}^{+}\right)$ possesses a non-zero transition dipole moment. In contrast, the transition dipole moment to the second excited state $S_{2}\left(\Sigma_{u}^{-}\right)$ is zero.

### 3.2 | Correspondence Between D(r) and Hamiltonian Matrix

If the external potential does not contain a spin-dependent operator, the Hamiltonian ${\hat{H}}_{v}$ simultaneously commutes with the total spin operator ${\hat{S}}^{2}$ and its projection ${\hat{S}}_{z}$ along a given axis z, each eigenstate of the Hamiltonian can be characterized by the spin quantum numbers, $\left|S,M_{s}\right\rangle (S=0,\;1/2,\;1,\ldots$ and $M_{S}=-S,-S+1,\ldots ,S-1,S)$ [77, 78]. Moreover, the density operator $\overset{ˆ}{\rho }(r)$ also commutes with spin operators. Thus, the matrix density D(r) is independent of spin [17]. Therefore, the spin quantum numbers must be explicitly specified to characterize all states in the subspace $S_{v}^{N}$. In fact, if any eigenstate of the (2S+1)-spin manifold is in the subspace, $\Psi_{S;M_{S}}\in S_{v}^{N}$, all multiplets of degenerate states must also be in the subspace: $\left\{\Psi_{S;M_{s}};M_{s}=-S,\ldots ,+S\right\}\in S_{v}^{N}$. Note that all degenerate multiplets have the same density and null transition densities.

In the previous section, we introduced the N-matrix density D(r), constructed from a set of states $\left\{\Phi_{A}\right\}$ where A=1,⋯,N. The same set of states also defines a Hamiltonian matrix $H=\left\{H_{AB};A,B=1,\cdots ,N\right\}$ via Equation (5). Utilizing tools in functional analysis [71, 79], a rigorous foundation for DFT applicable to multiple excited states has been established [17, 68]. Considering N eigenstates (with N>1) of the Hamiltonian ${\hat{H}}_{v}$, we have proven a theorem asserting the existence of a *Hamiltonian matrix functional* dependent on D(r) [17]:

#### Theorem 1.

Given the number of states N>1, there exists a one-to-one correspondence between the N-matrix density and the Hamiltonian matrix within the unique N-dimensional subspace $S_{v}^{N}$.

We prove Theorem 1 by *reductio ad absurdum*: Assume that within the subspace $S_{v}^{N}$, there exist two sets of linearly independent basis states, $\left\{\Phi_{A}\right\}$ and $\left\{\Phi_{B}^{\prime }\right\}$ related by the unitary matrix U, that give the same matrix density, $D(r)=D^{\prime }(r)$, but different Hamiltonian matrices, $H^{\prime }\neq H$. In other words, there exists a unitary matrix U, such that

(19) $$D^{\prime }(r)=U^{†}D(r)U=D(r),\;\;for\;all\;r\in R^{3}$$

There are only two possibilities that Equation (19) holds:

1. The unitary matrix U is the identity matrix I, in which case the two basis states $\left\{\Phi_{A}\right\}$ and $\left\{\Phi_{B}^{\prime }\right\}$ are identical and, thus, give the same Hamiltonian matrix, $H^{\prime }=H$;
2. The unitary matrix U only contains an arbitrary rotation within the same spin manifold, $V_{S}^{K}=span\left\{\Psi_{S,M_{s}}^{K};M_{s}=-S,\ldots ,+S\right\}$, where all $\Psi_{S,M_{s}}^{K}$ are degenerate eigenstates of ${\overset{ˆ}{H}}_{v}$ with the energy $E_{K},\;{\hat{H}}_{v}\Psi_{S,M_{s}}^{K}=E_{K}\Psi_{S,M_{s}}^{K}$. The spin manifold is a subspace of $S_{v}^{N},\;V_{S}^{K}\subset S_{v}^{N}$. Since all states in the spin manifold $V_{S}^{K}$ give an identical density and zero transition density, the overall matrix density within $V_{S}^{K}$, under any unitary transformation, is $\rho_{K}(r)*I$, proportional to the identity matrix I. Moreover, the Hamiltonian matrix within $V_{S}^{K}$ is $E_{K}*I$, also proportional to I. Therefore, the resulting Hamiltonian matrices are identical,

(20) $$H^{\prime }=U^{†}\left(E_{K}*I\right)U=E_{K}*I=H$$

In both scenarios, the Hamiltonian matrices are the same, $H^{\prime }=H$, contradictory to the initial assumption. Consequently, a given matrix density D(r) must correspond to a unique Hamiltonian and we have proved the one-to-one correspondence between the matrix density and the Hamiltonian matrix within $S_{v}^{N}$. This correspondence is defined as the Hamiltonian *matrix functional* of the matrix density, ℋ[D].

In general, if the subspace $S_{v}^{N}$ contains multiple spin manifolds, other than singlets (which is non-degenerate), the unitary matrix U that gives Equation (19) can be a product of multiple unitary matrices that represent rotations within different spin manifolds. The above statement still holds true.

We shall remark that in contrast to the unique ground-state density in KS-DFT, the matrix density D(r) in $S_{v}^{N}$ is not unique. From the above proof, one sees that the pair of the matrix density and the Hamiltonian matrix is only determined up to an arbitrary N-dimensional unitary matrix. Minded reader may also notice that the trace of D(r) and that of H, that is, the subspace density $\rho_{S}(r)$ and subspace energy $E_{N}$, remain unchanged with respect to the unitary transformation. Of course, the corresponding matrix elements of the two sets of matrices are different. This is a key difference between the ground-state theory and the multistate theory.

### 3.3 | Universal Matrix Functional

Given that the subspace energy is a functional of the external potential v(r), $E_{N}[v]$, we establish the existence of the universal matrix functional of N states, ${ℱ}_{N}[D]$, using techniques in functional analysis. The proof provides a solid mathematical foundation for DFT of multiple states [68]. First, we note that the supremum of the scalar subspace functional in Equation (13) indicates that there exists a sequence of external potentials, $v_{j}(r);j=1,2,\cdots$, such that

(21) $$F_{N}\left[\rho_{S}\right]=\underset{j\to \infty }{min}\;\{E_{N}\left[v_{j}\right]-\int drv_{j}(r)\rho_{S}(r)\}$$

Any potential within the sequence, $v_{j}(r)$, uniquely determines an N-dimensional subspace $S_{v_{j}}^{N}$. Then, from an arbitrary basis vector $\left\{\Phi_{A}^{v_{j}};A=1,\cdots ,N\right\}$ of the subspace $S_{v_{j}}^{N}$, we denote its corresponding matrix density and the Hamiltonian matrix as $\left(D_{v_{j}}^{\left(0\right)}(r),H^{\left(0\right)}\left[v_{j}\right]\right)$. Then with a given matrix density D(r), we choose the special pair of N-matrix density and Hamiltonian matrix, $\left(D_{v_{j}}(r),H\left[v_{j}\right]\right)$ by minimizing the Frobenius $\left(L^{2}\right)$ norm between $D_{v_{j}}(r)$ and D(r) such that the difference in the external potential energy is minimum. This can be accomplished via the unitary matrix $U_{j}$,

(22) $$U_{j}=arg\;\underset{U_{j}}{min}\;{\left\|\int \;drv_{j}(r)\left(D(r)-U_{j}^{†}D_{v_{j}}^{\left(0\right)}(r)U_{j}\right)\right\|}_{2}$$

where the $L^{2}$-norm of a matrix M is defined as $\|M{\|}_{2}={\left(\Sigma_{A\geq B}{\left|M_{AB}\right|}^{2}\right)}^{1/2}$. Therefore, the special pair $\left(D_{v_{j}}=U_{j}^{†}D_{v_{j}}^{\left(0\right)}U_{j},H\left[v_{j}\right]=U_{j}^{†}H^{\left(0\right)}\left[v_{j}\right]U_{j}\right)$ is obtained for a given potential $\nu_{j}(r)$. It follows that a *universal matrix functional* ${ℱ}_{N}[D]$ can be rigorously defined by,

(23) $${ℱ}_{N}[D]=\underset{j\to \infty }{lim}\;\left\{H\left[v_{j}\right]-\int drv_{j}(r)D_{v_{j}}(r)\right\}$$

where the sequence of potential functions $\left\{v_{j};j=1,2,\ldots \right\}$ is determined by Equation (21) and $H\left[v_{j}\right]$ is the Hamiltonian matrix given by, $H\left[v_{j}\right]=U_{j}^{†}H^{\left(0\right)}\left[v_{j}\right]U_{j}$, with $U_{j}$ determined by Equation (22). Recall that unitary transformation does not change the trace of a matrix. Thus, the optimization of Equation (22) represents a rotation within the same subspace $S_{v_{j}}^{N}$. Nevertheless, this rotation is unique with respect to the given N-matrix density D(r) (see Reference [68] for details).

We remark that the concept of the matrix functional ${ℱ}_{N}[D]$ was originally introduced by the authors in 2022 [17], in which each element of the N-dimensional square matrix, ${ℱ}_{N,AB}[D](A,B=1,\ldots ,N)$, is a functional of the entire matrix density D(r) as a whole, rather than a functional of its element, that is, ${ℱ}_{N,AB}[D]\neq {ℱ}_{N,AB}\left[D_{AB}\right]$.

There is an exact relation between the universal matrix functional and the scalar universal subspace functional.


(24) $$F_{N}\left[\rho_{S}=N^{-1}tr(D)\right]=N^{-1}tr\left({ℱ}_{N}[D]\right)$$

That is, ${ℱ}_{N}[D]$ defines $F_{N}\left[\rho_{S}\right]$ uniquely and completely. However, the reverse is not possible unless Theorem 1 is applied, which requires the N-matrix density as its fundamental variable. In other words, the information content of the subspace DFT is limited and, critically, insufficient to determine the spectrum of eigenstates.

### 3.4 | Hamiltonian Matrix Functional and Variation Principle

With the universal matrix functional ${ℱ}_{N}[D]$ of Equation (23), the *Hamiltonian matrix functional* introduced in Section 3.2 can now be written as follows:

(25) $$ℋ[D]={ℱ}_{N}[D]+\int drv(r)D(r)$$

Equation (25) is an explicit expression of Theorem 1, that is, the one-to-one correspondence between the matrix density and the Hamiltonian matrix. Moreover, ℋ[D] is not only defined within the subspace $S_{v}^{N}$, but applies to all possible N-dimensional matrix densities [68].

Notice that Equation (25) has an appearance in analogy to the scalar energy functional of Equation (11) for the ground state $E_{1}[\rho ]$. However, it is critical to realize that for multiple states, the relationship is in the form of the full Hamiltonian matrix functional of the N-matrix density, which is the fundamental variable. The concept of a Hamiltonian matrix functional is a fundamental distinction from a scalar subspace functional (Equation 14). As seen immediately below, the availability of the full Hamiltonian matrix functional allows the energies and densities of each and all individual eigenstates to be rigorously determined.

Before we introduce the variational principle for many-states, we define the scalar *multistate energy functional* $E_{MS}[D]$ as the trace of the Hamiltonian matrix functional,

(26) $$E_{MS}[D]=N^{-1}tr(ℋ[D])=N^{-1}\sum_{A=1}^{N}{ℋ}_{AA}[D]$$

Since the Hamiltonian matrix functional can be diagonalized by a unitary matrix, its trace $E_{MS}[D]$ is invariant and equal to the average of the eigenvalues of ℋ[D] in Equation (2). Therefore, the multistate energy functional has the same properties of the subspace energy. Hence, we use the term, *subspace energy functional*, interchangeably in reference to the same physical quantity.

#### Theorem 2.

(Variation principle of MSDFT) Minimization of the multistate energy functional $E_{MS}[D]$ with respect to a trial matrix density $D^{\prime }(r)$ leads to the exact subspace energy $E_{N}[v]$.

(27) $$E_{MS}\left[D^{\prime }\right]\geq E_{N}[v]=\underset{D(r)}{min}\;\left\{E_{MS}[D]\right\}$$

where the equal sign holds true when the optimal matrix density is exactly that of the subspace $S_{v}^{N}$, denoted as $D^{o}(r)$. Moreover, by inserting $D^{o}(r)$ into the Hamiltonian matrix functional ℋ[D], the eigenvalues of $ℋ\left[D^{o}\right]$ are exactly the N lowest eigenenergies of the system, $\left\{E_{1},\ldots ,E_{N}\right\}$.

We leave the details of the proof of Theorem 2 to reference [17].

One may wonder why the optimization of a scalar multistate energy functional will yield the exact energies (and densities) of all eigenstates of the N-dimensional subspace $S_{v}^{N}$. Indeed, the subspace variation principle is established upon subspace properties, employing a scalar, subspace-invariant variable, the subspace density $\rho_{S}(r)$ [74]. As a result, only the subspace energy and subspace density can be directly obtained, and there is insufficient information relating to the individual eigenstates (see discussion following Equation 24). It may be suggested that a non-uniform weighting of the individual states of the subspace might lead to the extraction of state properties by functional derivatives. However, there is no guarantee that eigenstates are in fact obtained, unless the exact eigenstate wave functions (not densities) are known in the first place (Equation 3). Further discussion is beyond the scope of this review.

On the other hand, the variation principle of MSDFT (Equation 27) is built on a subspace (multi-state) energy functional that is constructed by a Hamiltonian matrix functional with respect to the matrix density. Accordingly, variational minimization of the multi-state energy functional $E_{MS}[D]$ simultaneously optimizes the Hamiltonian matrix functional ℋ[D] and the matrix density D(r). Once the variational minimum is achieved, the optimized $ℋ\left[D^{o}\right]$ and the optimal matrix density $D^{o}(r)$ correspond to the subspace $S_{v}^{N}$, which is spanned by the N lowest eigenstates of the system, $\left\{\Psi_{I}\right\}$. Henceforth, diagonalization of the optimized $ℋ\left[D^{o}\right]$ gives the exact eigenenergies of $\left\{\Psi_{I}\right\}$,

(28) $$ℋ\left[D^{o}\right]C=Cℰ$$

where $ℰ=diag\left(E_{1},\ldots ,E_{N}\right)$ is a diagonal matrix of eigenenergies and C is the corresponding coefficient matrix. Then, the exact density of each eigenstate $\Psi_{I}$ (without knowing the exact many-body wave function) is given by

(29) $$\rho_{I}(r)=\sum_{AB}^{N}C_{AI}^{*}C_{BI}D_{AB}^{o}(r)$$

And, the energy functional of the exact eigenstate $\Psi_{I}$ is

(30) $$E_{I}[D]=\sum_{AB}^{N}C_{AI}^{*}C_{BI}{ℋ}_{AB}[D]$$

The theorems of existence of the Hamiltonian matrix functional and the variation principle (Theorems 1 and 2) establish the theoretical foundation of the multi-state DFT.

### 3.5 | Symmetry of the Hamiltonian Matrix Functional

Symmetry of the Hilbert space must be preserved in DFT. We define a *symmetry for the Hamiltonian matrix functional* as an operation 𝒪 to the matrix density, $𝒪:D(r)\to D^{\prime }(r)$, that leaves the subspace energy functional invariant,

(31) $$E_{MS}[D]=E_{MS}\left[D^{\prime }\right]$$

Given an arbitrary N-dimensional subspace $V^{N}\subset H$, we choose two sets of its orthonormal basis states $\left\{\Phi_{A};A=1,\ldots ,N\right\}$ and $\left\{\Phi_{B}^{\prime };B=1,\ldots ,N\right\}$, which are related by the N-dimensional unitary matrix $U,\Phi_{B}^{\prime }=\sum_{A=1}^{N}U_{AB}\Phi_{A}$. Then their corresponding matrix densities are related by the unitary transformation,

(32) $$D(r)\to D^{\prime }(r)=U^{†}D(r)U$$

where $D_{AB}(r)=\left\langle \Phi_{A}\right|\overset{ˆ}{\rho }(r)\left|\Phi_{B}\right\rangle$ and $D_{AB}^{\prime }(r)=\left\langle \Phi_{A}^{\prime }\right|\overset{ˆ}{\rho }(r)\left|\Phi_{B}^{\prime }\right\rangle$. Since it is completely equivalent to represent the Hamiltonian matrix within the subspace $V^{N}$ with either $\left\{\Phi_{A};A=1,\ldots ,N\right\}$ or $\left\{\Phi_{B}^{\prime };B=1,\ldots ,N\right\}$, the Hamiltonian matrix functional ℋ[D] must respect the following transformation under the unitary transformation of basis states,

(33) $$ℋ\left[U^{†}DU\right]=U^{†}ℋ[D]U$$

On the other hand, since the subspace energy functional is proportional to the trace of the Hamiltonian matrix functional (Equation 26), it is invariant under the unitary transformation,

(34) $$E_{MS}\left[U^{†}DU\right]=E_{MS}[D]$$

Henceforth, the unitary transformation of the matrix density (32), *induced* by the unitary transformation of basis states within the same subspace, is a symmetry of the Hamiltonian matrix functional. The transformation property (33) of ℋ[D] is a necessary condition for state interaction within a Hilbert space, which is required for the determination of individual eigenstate energies. This highlights a conceptual departure from the traditional Kohn–Sham approach to DFT for the ground state, in which a single state bears no state interaction [2].

## 4 | Method

The enormous success of Kohn–Sham density functional theory (KS-DFT) is due in no small part to the use of a non-interacting reference system with a single-determinant wave function to represent exactly the density of the ground state. While it is tempting to extend this method to multiple electronic states, the densities of multiple states cannot be captured using a single determinant for each state from a common set of orthonormal orbitals. The orbitals representing the density of each excited state must be relaxed if a single determinant is used. Further, to unequivocally determine the energies of eigenstates, state interactions are necessary, which require both state and transition densities. In this section, we present the conditions and theoretical limits for density representation of multiple electronic states. Based on this theoretical foundation, we introduce the concept of MAS, which naturally leads to the definition of correlation matrix functional in multistate DFT.

### 4.1 | Minimal Active Space

#### 4.1.1 | Theorems of Density Representation

In our original paper introducing Hamiltonian matrix density functional, we also proved a theorem on the representation of the N-matrix density D(r) using nonorthogonal orbitals [17]. This is further extended to the use of a common set of orthonormal orbitals [76]. In either case, an upper bound exists in the number of determinants needed to exactly represent D(r), providing a theoretical limit on the computational scaling in excited-state calculations.

##### Theorem 3.

An arbitrary N-matrix density D(r) can be sufficiently represented by $N^{2}$ nonorthogonal Slater determinants.

The proof of this theorem is rather straightforward: Starting with two arbitrary states $\Psi_{I},\;\Psi_{J}\in H$, with state densities $\rho_{I}(r)$ and $\rho_{J}(r)$, respectively. We construct two new states as linear combinations of $\Psi_{I}$, and $\Psi_{J}:\Phi_{+}=\left(\Psi_{I}+\Psi_{J}\right)/\sqrt{2}$, and $\Phi_{-}=\left(\Psi_{I}+i\Psi_{J}\right)/\sqrt{2}$. The corresponding densities of these two new states are given as $\rho_{\pm }(r)=\left\langle \Phi_{\pm }\right|\hat{\rho }(r)\left|\Phi_{\pm }\right\rangle$. Then, the transition density between states $\Psi_{I}$ and $\Psi_{J},\rho_{IJ}(r)$, can be obtained by expanding and rearranging the terms of $\rho_{\pm }(r)$. Specifically, the real part of the transition density is $ℛ\rho_{IJ}=\rho_{+}-\left(\rho_{I}+\rho_{J}\right)/2$ and the imaginary part is $ℱ\rho_{IJ}=\left(\rho_{I}+\rho_{J}\right)/2-\rho_{-}$. Since any state density can be mapped to a single Slater determinant [80, 81], the diagonal and off-diagonal elements of the 2-matrix density can be exactly represented by four independent determinants, respectively, for $\rho_{I}(r)$, $\rho_{J}(r)$, $\rho_{+}(r)$ and $\rho_{-}(r)$. In general, for any N-matrix density, there are N diagonal terms, and N(N−1) off-diagonal terms, for a total of $N^{2}$ determinants to fully represent D(r).

1. *Nonorthogonal representation*: Following Theorem 3, each of the $N^{2}$ elements, including generally the real and imaginary parts of transition densities, of an N-matrix density is mapped to a Slater determinant, which is independently optimized. These determinants are generally nonorthogonal.
2. *Orthogonal representation*: Alternatively, an N-matrix density D(r) can also be represented by a set of determinants constructed based on a set of *orthonormal orbitals*.

##### Corollary 1.

(Orthogonal representation of D(r)): For an N-matrix density D(r), it is always possible to find a set of orthonormal orbitals, $A_{o}=\left\{\psi_{p};p=1,\cdots ,n_{A}\right\}$ such that each element⋯ $D_{AB}(r)$ of D(r) can be represented by $\left\{\psi_{p}\right\}$ for all A,B=1,⋯,N, where $n_{A}\leq (4N-3)Nn,\;n$ is the number of electrons and N is the number of eigenstates of interest.

These orbitals are called *active orbitals*. Then, the number of determinants needed to represent D(r) exactly is in the order of $𝒪\left(n^{2}n_{A}^{2}\right)$. Details of the proof can be found in Reference [76].

#### 4.1.2 | Minimal Active Space

Whether a set of orthonormal orbitals is used or nonorthogonal determinants are adopted, there is an upper bound in the number of determinants to exactly represent the N-matrix density. Clearly, if real densities are used, the number of determinants needed to represent the matrix density will be considerably smaller. In principle, determinants can be directly used to map elements of a matrix density; however, equivalently, it is convenient to formulate a set of N auxiliary multi-configurational wave functions, $\left\{\Omega_{A};A=1,\cdots ,N\right\}$, in terms of these determinants.

Specifically, the existence of an upper bound presented in Theorem 3 and Corollary 1 defines a MAS, consisting of no more than $N^{2}$ Slater determinants to exactly represent an N-matrix density D(r), if nonorthogonal determinants are used (Theorem 3):

(35) $$V_{MAS}^{N}=span\left\{\Xi_{\zeta };\zeta =1,\ldots ,N^{2}\right\}$$

Here, each determinant is constructed by n orthonormal spin orbitals (n being the number of electrons), $\Xi_{\zeta }=det\left\{\phi_{1}^{\zeta }\ldots \phi_{n}^{\zeta }\right\}$, where $\left\langle \phi_{j}^{\zeta }|\phi_{k}^{\zeta }\right\rangle =\delta_{jk}$, but, in general, $\left\langle \phi_{j}^{\zeta }|\phi_{m}^{\eta }\right\rangle \neq 0$.

In case where a common set of orthonormal orbitals is used (Corollary 1), the MAS $V_{MAS\;}^{N}$ is spanned by a reference determinant $\Xi_{0}=det\left\{\psi_{1}\ldots \psi_{n}\right\}$ made of n active orbitals from $A_{o}$ and its singly and doubly excited determinants, $\left\{\Xi_{j}^{a};j=1,\ldots ,n,a=(n+1),\ldots ,n_{A}\right\}$ (excitation of an electron from orbital $\psi_{j}$ to $\psi_{a}$) and $\left\{\Xi_{jk}^{ab};j,k=1,\ldots ,n;a,b=(n+1),\ldots ,n_{A}\right\}$ (two-electron excitation from orbitals $\psi_{j},\;\psi_{k}$ to $\psi_{a},\;\psi_{b}$). That is,

(36) $$V_{MAS\;}^{N}=span\left\{\Xi_{0},\Xi_{j}^{a},\Xi_{jk}^{ab};j,k=1,\ldots ,n;a,b=(n+1),\ldots ,n_{A}\right\}$$

Then, the N auxiliary wavefunctions are expressed as linear combinations of the determinant basis states in $V_{MAS}^{N}$:

(37) $$\Omega_{A}=\sum_{\zeta =1}^{M}c_{\zeta }^{A}\Xi_{\zeta };A=1,\ldots ,N$$

where $M\leq N^{2}$ if nonorthogonal determinants are used, and $M=O\left(n^{2}n_{A}^{2}\right)$ if a common set of orthonormal orbitals is used. For convenience, a single index is used to label determinant configurations. The element of the N-matrix density D(r) can be exactly represented as follows:

(38) $$D_{AB}(r)=\left\langle \Omega_{A}\right|\overset{ˆ}{\rho }(r)\left|\Omega_{B}\right\rangle$$

We note that Theorem 3 does not give a precise way of selecting and constructing the specific configurations in the MAS. Rather, it proved the mathematical bound for a finite number of determinants that is sufficient to represent the N-dimensional matrix density. In order to determine the exact D(r) to construct the Hamiltonian matrix functional, additional conditions are needed, which are of interest for future investigations. Nevertheless, Theorem 3 and the concept of MAS provide the theoretical foundation for developing computational models in practice, two of which are described below.

### 4.2 | The Correlation Matrix Functional

The introduction of MAS to construct a set of auxiliary multiconfigurational wave functions to exactly represent D(r) naturally leads to the definition of the correlation matrix functional and design of different computational algorithms [76, 82].

On the basis of the MAS for matrix density representation, an explicit representation of ${ℱ}_{N}[D]$ can be obtained via the Hamiltonian matrix functional ℋ[D] of Equation (25). Specifically, given an arbitrary N-matrix density D(r), represented using either non-orthogonal or orthogonal determinants, we look for a unique set of auxiliary wavefunctions $\left\{\Omega_{A}^{min};A=1,\ldots ,N\right\}$, whose Hamiltonian matrix with respect to ${\hat{H}}_{0}$ yields the minimal trace. This can be accomplished by searching over all possible MASs in which there exist a set of auxiliary wavefunctions $\left\{\Omega_{A};A=1,\ldots ,N\right\}$ that give rise to D(r),

(39) $$\left\{\Omega_{A}^{min}\right\}=arg\;\underset{\left\{\Omega_{A}\right\}}{min}\;\left\{tr\left(F\left[\left\{\Omega_{A}\right\}\right]\right)|\left\{\Omega_{A}\right\}\to D(r)\right\}$$

where the matrix $F\left[\left\{\Omega_{A}\right\}\right]$ is defined with the element, $F_{AB}\left[\left\{\Omega_{A}\right\}\right]=\left\langle \Omega_{A}\right|{\overset{ˆ}{H}}_{0}\left|\Omega_{B}\right\rangle$ for all A,B=1,…,N.

Consequently, we define the *correlation matrix functional*, denoted by ${ℰ}^{c}[D]$, as the difference between the universal matrix functional ${ℱ}_{N}[D]$ and the projection of ${\hat{H}}_{0}over\left\{\Omega_{A}^{min};A=1,\ldots ,N\right\}$, whose element is given by

(40) $${ℰ}_{AB}^{c}[D]={ℱ}_{N,AB}[D]-\left\langle \Omega_{A}^{min}\right|{\overset{ˆ}{H}}_{0}\left|\Omega_{B}^{min}\right\rangle$$

for all A,B=1,…,N. We shall point out that since state interactions are accounted for among $\left\{\Omega_{A}^{min}\right\}$, a portion of electronic correlation has been captured by the Hamiltonian matrix of $\left\{\Omega_{A}^{min}\right\}$. The remaining part of electronic correlation is incorporated into the correlation matrix functional ${ℰ}^{c}[D]$.

### 4.3 | Exact Properties of Correlation Matrix Functional

The correlation matrix functional is defined with respect to the MAS on the basis of the universal matrix functional (40). The most important task now is to construct effective approximations of ${ℰ}^{c}[D]$.

The unitary transformation of the Hamiltonian matrix under basis transformation within a finite Hilbert space is trivially understood in the wavefunction theory. In DFT, the symmetry of the Hamiltonian matrix functional (33) imposes a critical constraint on the analytical structure of the correlation matrix functional, which must be transformed identically [83]:

(41) $${ℰ}^{c}\left[U^{†}DU\right]=U^{†}{ℰ}^{c}[D]U$$

The relation is significant because ${ℰ}^{c}[D]$ is in general a non-linear functional of the matrix density D(r). As a result, Equation (41) establishes an equality between a nonlinear expression of U on the left with a bilinear expression on the right, which holds true for any N-dimensional unitary matrix U. Equations (33), (34), and (41) together are named the *subspace invariance property*, a critical symmetry that guarantees correct state interactions within the Hilbert subspace.

#### 4.3.1 | Theorem of Correlation Matrix Functional

From the subspace invariance property (41), a fundamental relationship that is universal for all possible matrix densities is obtained between the diagonal and off-diagonal elements of ${ℰ}^{c}[D]$. It defines the structure of the correlation matrix functional.

##### Theorem 4.

Given any state correlation functional, that is, one diagonal term of ${ℰ}^{c}[D]$, all elements of the correlation matrix functional in an N-dimensional Hilbert subspace are uniquely determined.

To prove this theorem, we applied techniques of Lie groups and Lie algebras [84, 85] on the basis of the subspace invariance property of the correlation matrix functional (41). Details on the derivation can be found in reference [83].

## 5 | Algorithm

Thus far in this review, the fundamental theory, concepts, and methods underlying MSDFT have been established with mathematical rigor and supported by comprehensive mathematical proofs. Practical computations using MSDFT necessitate introducing approximations—most notably to the correlation matrix functional—as well as making choices regarding the MAS and specific optimization algorithms. In the following section, we present several approaches that have been successfully explored and applied to a wide range of ground-state and excited-state problems in chemical and biological systems.

### 5.1 | Multi-State Self-Consistent Field Theory (MSSCF)

**FIGURE 2 |:**
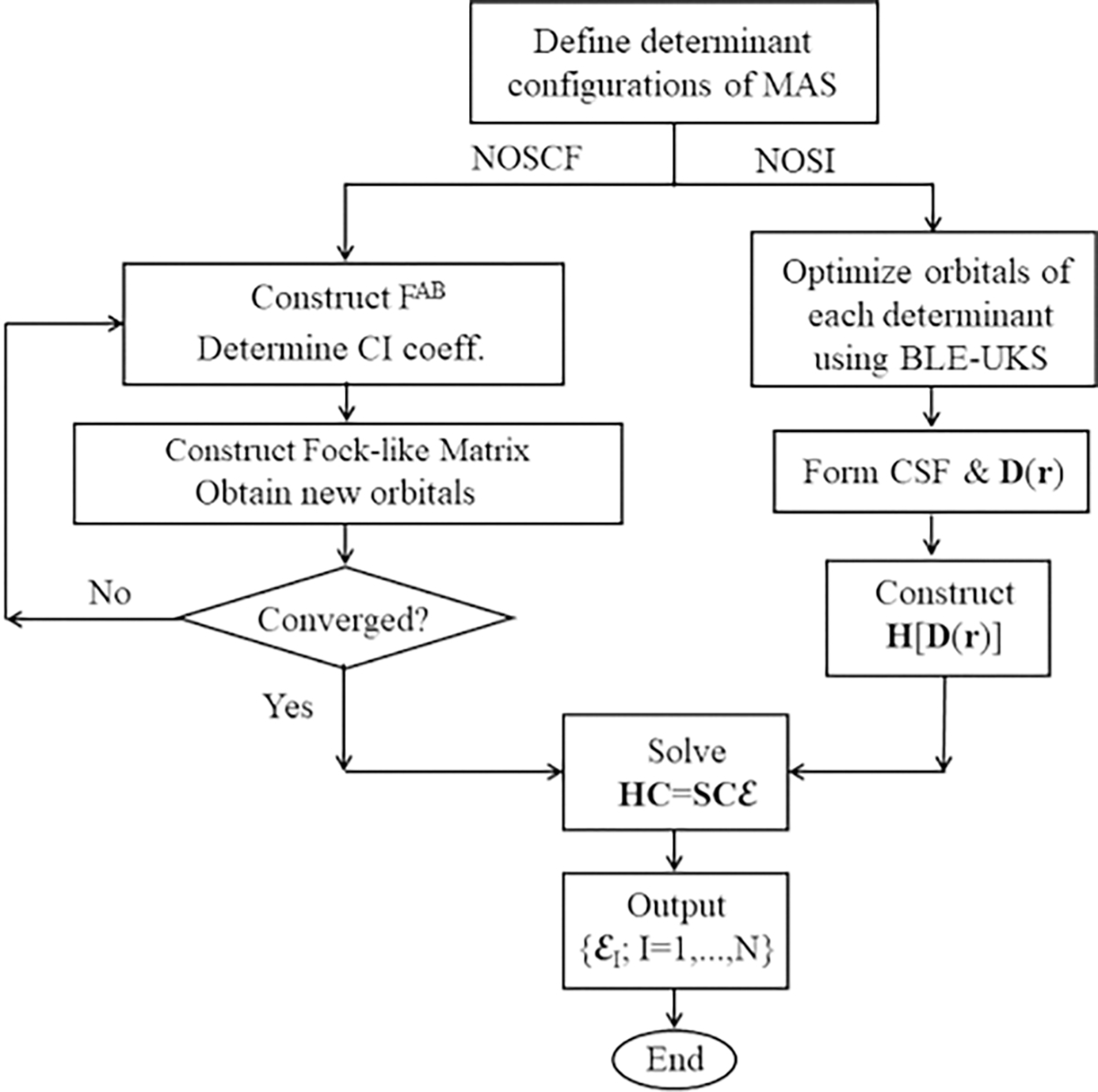
Flowchart illustrating key computational steps for the nonorthogonal self-consistent-field (NOSCF) and nonorthogonal state interaction (NOSI) methods. Reproduced from reference [82] with permission by the American Chemical Society.

MSDFT computation can be carried out by self-consistent-field optimization of both orbital and state-configuration coefficients of the auxiliary multiconfigurational wave-functions of Equation (37) for representing the N-matrix density D(r); this is denoted as MSSCF. Alternatively, MSDFT can be performed by using a nonorthogonal state interaction (NOSI) algorithm. The key computational steps are illustrated in the flowchart in Figure 2 [82].

We use the term multistate SCF (MS-SCF) to distinguish the present DFT from the well-known MC-SCF methods in WFT because the basis states in the active space for MSDFT include dynamic correlation in the first place, belonging to a dynamic-and-static ansatz. In MSSCF, we minimize the multistate energy (Equation 27) subject to the constraints of normalization for each multiconfigurational state $<\Omega_{A}|\Omega_{B}>=\delta_{AB}$ (Equation 37), and the orthonormal conditions for orbitals within each determinant, $<\phi_{i}^{\zeta }|\phi_{j}^{\zeta }>=\delta_{ij}$ (Equation 35). We introduce the Lagrangian

(42) $$L[D]=E_{MS}[D]-N^{-1}\sum_{A}^{N}\;E_{A}\left(\sum_{\xi \zeta }^{N^{2}}\;c_{\xi }^{A}S_{\xi \zeta }c_{\zeta }^{A}-1\right)\;\;-\sum_{\xi }^{N^{2}}\;\sum_{\sigma =\alpha ,\beta }\;\sum_{jk}^{n_{\sigma }}\;\epsilon_{jk;\sigma }^{\xi }\left(\left\langle \phi_{j\sigma }^{\xi }|\phi_{k\sigma }^{\xi }\right\rangle -\delta_{jk}\right)$$

where $\left\{E_{A}\right\}$ and $\left\{\epsilon_{jk;\sigma }^{\xi }\right\}$ are the Lagrange multipliers with respect to the normalization conditions and $S_{\xi \zeta }=<\Xi_{\xi }|\Xi_{\eta }>$ is the overlap between two nonorthogonal determinants. Minimization of L[D] with respect both to $c_{\xi }^{A}$ and $\phi_{j\sigma }^{\xi }$ leads to a set of coupled Fock-like self-consistent-field equations, respectively for the configurational coefficients and orbital coefficients. Since we have not implemented the MSSCF procedure, we ask interested reader to check reference [82] for the details. To this end, we note that the nonorthogonal SCF (NO-SCF) method and software developed by Lee Thompson’s group are especially relevant and, in principle, the present MSDFT could be adapted to that program [86–88]. In the following, we focus on the NOSI procedure, which has been implemented and applied to a range of problems of chemical and biological interest.

### 5.2 | NOSI

The computational procedure of NOSI is closely related to the well-known nonorthogonal configuration interaction (NOCI) in wave function theory [89–91], except that dynamic correlation is included in orbital optimization for each determinant configuration. The concept of NOSI was described by Liu and coworkers [57, 60] and it was used in the work of references [92, 93], and it has been refined and used in a range of applications to determine the energies of ground and excited states. In practice, it is convenient to express the total Hamiltonian and correlation matrix functionals (Equation 25) in terms of determinant configurations of $V_{MAS}^{N}$. Then, the Hamiltonian matrix density functional on a determinant basis is explicitly given by

(43) $$H_{\zeta \eta }[D]=<\Xi_{\zeta }|\overset{ˆ}{H}|\Xi_{\eta }>+E_{\zeta \eta }^{c}[D]$$

Notice that we have switched to standard Greek scripts for the elements of matrix functionals in determinant basis.

The NOSI computational procedure involves two main steps:

1. *Optimization of Individual Determinants*: Each determinant $\Xi_{\zeta }(\zeta =1,\cdots ,M)$, representing ground or excited configurations within the active space, is optimized separately (see below). The number of determinants (M) in the MAS may vary and is not strictly limited to $N^{2}$, where N is the number of states of interest, as it depends on the inclusion of all spin-complementary configurations and diabatic states under consideration. Because individual excited states are optimized, it limits the application of NOSI to low-lying excited states where occupied orbitals contributing to these states have adequate separations from higher virtual orbitals.
2. *Multistate Interaction*: After obtaining the optimized determinants, the energies and auxiliary wave functions (or densities) of the individual eigenstates are determined by diagonalizing the Hamiltonian matrix functional spanned by the determinant basis, HC=SCℰ. This step allows for the calculation of eigenstate energies and provides insights into electronic structures of systems such as local and charge-transfer interactions.

### 5.3 | Occupation-Constrained Orbital Optimization

The key in MSDFT calculation, both using nonorthogonal SCF and NOSI, is to select excited configuration states, that is, MAS, and to optimize their orbitals. The NO-MSSCF approach couples the optimizations of configuration-state and orbital coefficients, whereas the latter (NOSI) is straightforward by treating each determinant configuration separately. The main issue is to ensure that the character of the non-aufbau occupied orbitals in an excited state configuration remains unchanged without collapsing to the ground state or a lower energy state during SCF iteration. To this end, numerous methods have been described aimed at solving such ΔSCF solutions both within the Hartree–Fock and Kohn–Sham DFT frameworks [94–108]. We have developed two alternative approaches, namely block-localized excitation (BLE) and target state optimization (TSO) [109–111]. These methods can be used for not only a fully delocalized molecular system but also a treat of excited states within an individual fragment (local excitation) or across different blocks (charge transfer).

#### 5.3.1 | Block-Localized Kohn–Sham Orbitals

We start with the optimization of block-localized Kohn–Sham (BLKS) orbitals for the ground state of different molecular fragments in a common system [112]. In this approach, originally developed in block-constrained Hartree–Fock calculations for energy decomposition analysis [113, 114] the total Slater determinant wavefunction is constructed from BLKS orbitals [112]. Then, a block-localized determinant is written as follows,

(44) $$\Xi_{\eta }=K_{\eta }\hat{A}\left\{\Theta_{\eta }^{1}\Theta_{\eta }^{2}\cdots \Theta_{\eta }^{B}\right\}$$

where $K_{\eta }$ is a normalization constant, $\hat{A}$ is the antisymmetrizer, B is the number of fragmental blocks of the system, and $\Theta_{\eta }^{k}$ represents a product of BLKS spin-orbitals $\left\{\phi_{j\eta }^{k}\right\}$ localized in fragment block $k,\Theta_{\eta }^{k}=\phi_{1\eta }^{k}\phi_{2\eta }^{k}\cdots \phi_{n_{k}\eta }^{k}$ with $n_{k}$ being the number of electrons in fragment k.

Methods for optimizing such fragmental-block localized orbitals date back to the works of Stoll et al. [115]; it was first used by Mo and coworkers in KS-DFT calculations [112]. The energy expression for the block-localized system has an identical form as that of standard KS-DFT [92]:

(45) $$E_{\eta }^{BLKS}=tr(Ph)+\frac{1}{2}tr(PJP)+E_{\eta }^{xc}\left[\rho_{\eta }\right]$$

where $P=C{\left(C^{†}SC\right)}^{-1}C^{†}$ is the generalized density matrix for nonorthogonal BLKS orbitals with S being the basis orbital overlap matrix and C the orbital coefficient matrix. In Equation (45), h and J are the conventional one-electron and two-electron Coulomb integrals, and $E_{\eta }^{xc}$ is the Kohn–Sham exchange-correlation energy.

#### 5.3.2 | Block-Localized Excitation

BLE is a form of ΔSCF optimization technique. One small step was taken to constrain excitations within a specified fragment block or across different blocks with respect to BLKS orbitals for the ground state (Equation 44). However, one giant benefit is its ability to define, as rigorously as possible, local excitations of one fragment in the presence of other fragments in the ground state or charge-transfer states between different fragments. In short, BLKS for the ground state and BLE for excited states constrain localized charge and spin diabatic states in orbital space, in sharp contrast to other constrained DFT models that employ spatial confinement.

As all ΔSCF models, it is crucial to maintain a given non-aufbau occupation during optimization. In BLE, a preordered orbital projection scheme, in close analogy to the guided SCF method [98], is applied to preserve the ordering of orbitals. In particular, we solve the eigenvalue problem, $(C^{o†}FC^{o})C=(C^{o†}{FC}^{o}){CE}^{BLE}$, effectively obtaining the excited non-aufbau orbitals (C) in the basis of the pre-ordered BLKS orbitals, ($C^{o†}{FC}^{0}$), from ground state calculations. With this projection, a preconditioning, it is not needed to resort the eigenvectors according to the energy of each orbital after diagonalization to solve for C as the order of ground state BLKS orbitals is kept.

The BLE procedure is generally applicable to any combinations of α and β spins for the excited electrons and multiple excitation can certainly be treated.

#### 5.3.3 | TSO

A common problem of ΔSCF calculations is the possibility of variational collapse to a lower energy state. To circumvent this problem from happening altogether, we place the unoccupied spin orbitals below the excited electron into a unique, separate block such that these orbitals will not be admixed with the remaining orbitals [110, 111]. As a result, the orbital in which the excited electron resides becomes the highest occupied orbital in the block that is being optimized. Consequently, the isolation of these intermediate orbitals effectively translates the optimization to a higher-energy solution of the Roothaan-Hall equation into a standard ground state optimization. Because of orthogonality conditions of the initial molecular orbitals used as the basis, there is no possibility that this TSO will collapse to states in which the isolated orbitals must be occupied.

**FIGURE 3 |:**
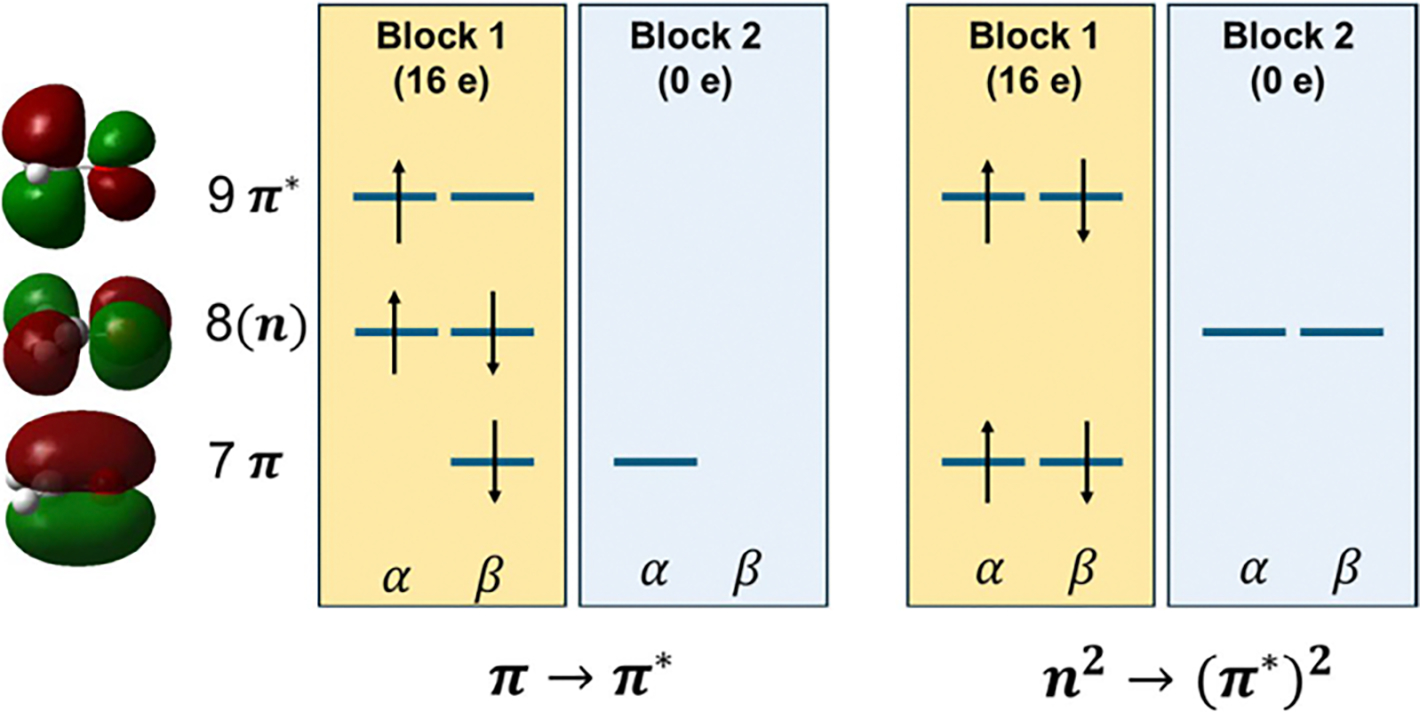
Orbital partition in the target-state optimization method illustrated for the singly $\pi \to \pi^{*}$ (left) and doubly $n^{2}\to {\left(\pi^{*}\right)}^{2}$ transitions of formaldehyde. The π(7), n(8), and $\pi^{*}$ orbitals are shown for a total of 16 electrons in the molecular orbital block 1 and the isolated orbital(s) in block 2 with zero electrons.

The TSO orbital-block partition is illustrated by the $\pi \to \pi^{*}$ excitation and the $n^{2}\to {\left(\pi^{*}\right)}^{2}$ double excitation of formaldehyde $\left({CH}_{2}C=O\right)$ in Figure 3 [111]. We first optimize the ground state, using unrestricted KS-DFT for convenience of discussion, in which Kohn–Sham spin orbitals up to 8α and 8β are occupied. Then, for the α-electron excitation $\pi \to \pi^{*}$, that is, HOMO-1→ LUMO, we isolate the HOMO-1 orbital into block 2. Figure 3 (left) depicts the two blocks, one active with all but the ground-state HOMO-1, $\pi^{\alpha }$ orbital and all 16 electrons, and another with an isolated “HOMO-1” having zero electrons. The TSO-SCF optimization is now reduced to a standard, ground-state BLKS optimization described in Section 5.3.1. Figure 3 (right) shows the double excitation $n^{2}\to {\left(\pi^{*}\right)}^{2}$ state, in which the two blocks contain, respectively, $n_{b}-2$ and 2 orbitals, where $n_{b}$ is the total number of basis functions.

For convenience, we can keep the number of α and β spin orbitals to be equal respectively in each block. Thus, in the first $\pi \to \pi^{*}$ case, we can place the highest-energy unoccupied β spin orbital in block 2. Details of the optimization procedure for nonorthogonal BLKS orbitals can be found in References [110, 111, 114].

### 5.4 | Correlation Matrix Functional Approximations

#### 5.4.1 | Kohn–Sham Correlation Approximation for the Diagonal Elements

As stressed above, there is, in general, no term-by-term correspondence between a matrix function variable and its matrix functional, for example, $D_{\zeta \eta }↔{\left(E_{c}\left[D_{\zeta \eta }\right]\right)}_{\zeta \eta }$. Rather, each element of a matrix functional depends on the entire matrix function variable: ${ℰ}_{\zeta \eta }^{𝒸}\equiv {\left({ℰ}^{𝒸}[D]\right)}_{\zeta \eta }$. However, in view of the remarkable successes and a whole range of approximate exchange-correlation functionals developed for KS-DFT, we can make the approximation for the diagonal elements of the correlation matrix functional:

(46) $${ℰ}_{\zeta \zeta }^{c}[D]\approx E_{c}\left[D_{\zeta \zeta }\right]$$

where on the right-hand-side, $E_{c}\left[D_{\zeta \zeta }\right]$ is a state correlation functional of the corresponding state density $\rho_{\zeta }\equiv D_{\zeta \zeta }$. Then, we find that the energy expression for KS-DFT can be substituted into the diagonal entry of the Hamiltonian matrix functional, given that the corresponding state densities are optimized by occupation-constrained BLKS-DFT except that for the ground state. This suggests that we may adopt one of the exchange-correlation functionals developed for Kohn–Sham theory to determine $H_{\zeta \zeta }$, making use of the non-aufbau occupation-constrained density as the input variable,

(47) $$H_{\zeta \zeta }\approx E^{BLKS}\left[D_{\zeta \zeta }\right]=<\Xi_{\zeta }|\overset{ˆ}{H}|\Xi_{\zeta }>+E_{c}^{KS}\left[\rho_{\zeta },\Xi_{\zeta }\right]$$

where $E^{BLKS}\left[D_{\zeta \zeta }\right]$ is the KS-DFT energy with the density $\rho_{\zeta }=D_{\zeta \zeta }$ optimized from the BLKS determinant $\Xi_{\zeta }$.

Here, we have defined the Kohn–Sham correlation energy $E_{c}^{KS}$ as the difference between $E^{BLKS}\left[D_{\zeta \zeta }\right]$ and Hartree–Fock energy using the same determinant and it is used to approximate the diagonal element of the correlation matrix functional:

(48) $$E_{c}^{KS}\left[\rho_{\zeta }\right]=E^{BLKS}\left[\rho_{\zeta },\Xi_{\zeta }\right]-E^{HF}\left(\Xi_{\zeta }\right)$$

#### 5.4.2 | Spin-Multiplet Degeneracy Constraint for Spin-Coupling

For the off-diagonal elements of the correlation matrix functional, we first consider spin coupling interactions which can be determined consistently, if not rigorously. In this case, we obtain the off-diagonal element, accounting for spin-coupling interactions, by enforcing energy degeneracy among spin multiplets with respect to the highest-spin configuration for which a single determinant is adequate and KS-DFT can be used [116, 117].

We begin with the interaction between two electrons, which yields a singlet state and a triplet (ST) state with $M_{S}=0$. The latter state is degenerate with the all spin-up configuration ($\Xi^{\left(↑↑\right)};\;M_{S}=1$). Then, by enforcing the degeneracy between the $M_{S}=0$ and $M_{S}=1$ multiplets of the triplet state, the element for the correlation matrix functional, ${\left(E_{c}\right)}_{\zeta \eta }^{ST}$, between the two determinant configurations $\Xi_{\zeta }^{\left(↑↓\right)}$ and $\Xi_{\eta }^{\left(↓↑\right)}$ can be rigorously determined by [116],

(49) $${\left(E_{c}\right)}_{\zeta \eta }^{ST}=E_{c}^{KS}\left[\rho^{\left(↑↑\right)},\Xi^{\left(↑↑\right)}\right]-E_{c}^{KS}\left[\rho_{\zeta }^{\left(↑↓\right)},\Xi_{\zeta }^{\left(↑↓\right)}\right]$$

where “KS correlation” energy is defined in Equation (48). In Equation (49), the spin-flip determinant $\Xi_{\eta }^{\left(↓↑\right)}=\left|\phi_{1\alpha }^{\eta }\cdots \phi_{(n/2-1)\beta }^{\eta }\phi_{i\beta }^{\eta }\phi_{j\alpha }^{\eta }\right|$ has an identical energy and can be equivalently used. Equation (49) implies that an extra KS-DFT calculation for the all spin-up configuration $\Xi^{\left(↑↑\right)}\left(M_{S}=1\right)$ is needed, but the extra computational time is negligible.

Similar expression for spin-coupling (sc) among three electrons in three orbitals for the doublet-quartet manifold can be derived [117, 118]. For interactions involving more than three electrons, $n_{sc}\geq 3$, the solution to the off-diagonal, spin-coupling elements will no longer be unique since there are more equations $\left(n_{sc}\left(n_{sc}+1\right)/2\right.$ off-diagonal elements) than energies differences with the all spin-up configuration $\left(n_{sc}\right)$. However, a solution that satisfies the condition that spin-multiplet degeneracy is preserved can be obtained by singular value decomposition [117].

#### 5.4.3 | Other Off-Diagonal Matrix Elements

The off-diagonal elements of ${ℰ}^{c}[D]$ for all other situations are determined by the overlap-weighted average correlation energy of the two interacting states.


(50) $${ℰ}_{\zeta \eta }^{c}[D]=\frac{1}{2}S_{\zeta \eta }\left(E_{c}^{KS}\left[\rho_{\zeta }\right]+E_{c}^{KS}\left[\rho_{\eta }\right]\right)$$

It turns out that it is the leading term in an expression that satisfies the symmetry invariance property in Equation (41) if the approximation of Equation (47) is used. This expression was proposed in the original application of MSDFT before its fundamental theorems were proved [92].

### 5.5 | *Qbics* Software: Quantum Biology, Information, and Chemical Sciences

Early computations were carried out using the package BLW-ED interfaced with the GAMESS-US program for electronic integral evaluation. The BLW-ED program was initially written by Yirong Mo for energy decomposition analysis using the block-localized wavefunction (BLW-ED) method [113] and for mixed molecular orbital and valence bond (MOVB) NOCI theory [91], for simulation of chemical and charge transfer reactions in condensed phases using combined quantum mechanical and molecular mechanical (QM/MM) potentials [119]. The QM/MM approach was initially implemented by Merek Frendorf into the Monte Carlo simulation package MCQUB developed by one of the authors. BLW-ED was later extended to block-localized Kohn–Sham DFT calculations, which combined with MOVB, resulted in the backbone for NOSI calculations [92]. The BLE and TSO, initially called generalized BLW, were developed by Peng Bao and Adam Grofe, respectively, who introduced many features into the BLW-ED program [109, 110]. BLW-ED was written in Fortran.

Since 2021, an effort led by Jun Zhang at Shenzhen Bay Laboratory resulted in the *Qbics* program (Quantum Biology, Informatics and Chemical Sciences) written in C++ [120]. *Qbics* is a comprehensive package for electronic structure calculations, molecular mechanics and modeling, and combined QM/MM molecular dynamics simulations. The MSDFT-NOSI method is one of the key features of *Qbics*, which is available at https://qbics.info.

## 6 | Applications

### 6.1 | Ground and Excited-State Energies

#### 6.1.1 | Ground-State Energies

A key issue in multiconfigurational approaches to DFT is the potential of double counting electron correlation. This problem is indeed encountered in methods that perform MCSCF calculations—such as CASSCF optimizations—followed by a dynamic correlation correction using a Kohn–Sham exchange-correlation energy:

(51) $$E_{g}^{\text{MC}-\text{DFT}}=E_{g}^{\text{CASSCF}}+E_{c}^{\text{KS}}\left[\rho_{g}^{\text{CASSCF}}\right]$$

Much have already been written on this problem with these models [63, 121]. On the other hand, the correlation energy associated with a MAS is fundamentally rigorous and well-defined in MSDFT. There is no double counting of the correlation in the matrix functional by the variational principle of Equation (27) for the N-dimensional subspace just as the Kohn–Sham exchange-correlation functional is defined for the Kohn–Sham determinant to represent the density of the ground state. Moreover, the correlation matrix functional distributes the total correlation energy among individual states.

In MSDFT-NOSI computations (hereafter simply referred to as NOSI), which use approximations of the correlation matrix functional, we address double counting of correlation by comparing the absolute electronic energies of ground states obtained using KS-DFT and NOSI with the same approximate functional. If KS-DFT yields the exact ground state energy (as it would when using the exact universal functional for the ground state), any double counting of the correlation in NOSI would result in a ground state energy significantly lower than the KS-DFT limit, assuming that the ground state is well described by a single reference wave function.

Table 1 lists the total energies of singlet and triplet ground states for a series of small compounds in the database of theoretical best estimates (TBE) constructed by Loos and coworkers [122] and a group of medium-sized organic species used in multistate energy decomposition analysis (MS-EDA) [123], along with the differences from KS-DFT results. For both groups of compounds, the M06-2x functional [124] was used coupled with the aug-cc-pVTZ basis functions [125] for the TBE dataset [126] and cc-pVDZ for the MS-EDA monomers [123]. We note that M06-2x is one of the functionals that includes much of the dispersion energy within the density functional approximation [124]. Thus, it is assumed that the method is transferrable to describing dispersion effects in the excited states. This is particularly advantageous since an empirical correction to dispersion energy for the ground state has no access to dispersion effects in excited states.

We find that for the singlet states of 28 molecules in Table 1, all but three have energy difference less than 0.001 a.u. relative to KS-DFT values. In fact, the overall root-mean-square difference (RMSD) is only 0.0006 a.u. (approximately 0.3 kcal/mol); excluding the three outliers, the mean root-square difference drops below 0.0003 a.u. The three molecules with larger deviations—ammonia, formaldehyde, and acetone at the $S_{1}$ excited state geometry—may exhibit multiconfigurational character and the results from MSDFT are in fact a realistic reflection of this effect.

The same trend in correlation effects on the lowest triplet-state energies is observed, although the RMSD for all 28 geometries is slightly higher at 0.008 a.u. than that of singlet states. Overall, the results presented in Table 1, coupled with additional studies not included here for brevity, demonstrate that double counting of correlation effects in MSDFT-NOSI calculations is negligible in comparison with the “exact” energies from KS-DFT, by employing the same KS approximate functional to estimate the correlation energy during single-determinant optimizations.

#### 6.1.2 | Excitation Energies

To illustrate the performance of NOSI on excited states, we compare the computed vertical excitation energies obtained using NOSI with those determined via wave function methods and linear-response time-dependent density functional theory (LR-TDDFT). We begin with a set of 18 small molecules, for which Loos and coworkers have meticulously established TBE of vertical excitation energies that approach the full CI limit [122]. Zhu et al. constructed a MAS for each molecule to determine the vertical excitation energies established in the TBE set [126]. Each MAS typically consisted of singly excited configurations involving 4 electrons from the two highest occupied orbitals to the two lowest unoccupied orbitals, whereas for diatomic molecules more configurations were included because of high symmetry and degenerate orbitals. For ammonia and nitrosomethane the HOMO-2,3 orbitals (four additional electrons) was also included and for the latter an additional $n^{2}\to {\left(\pi^{*}\right)}^{2}$ double excitation was included. Further details on the MAS are given in the original paper [126].

**FIGURE 4 |:**
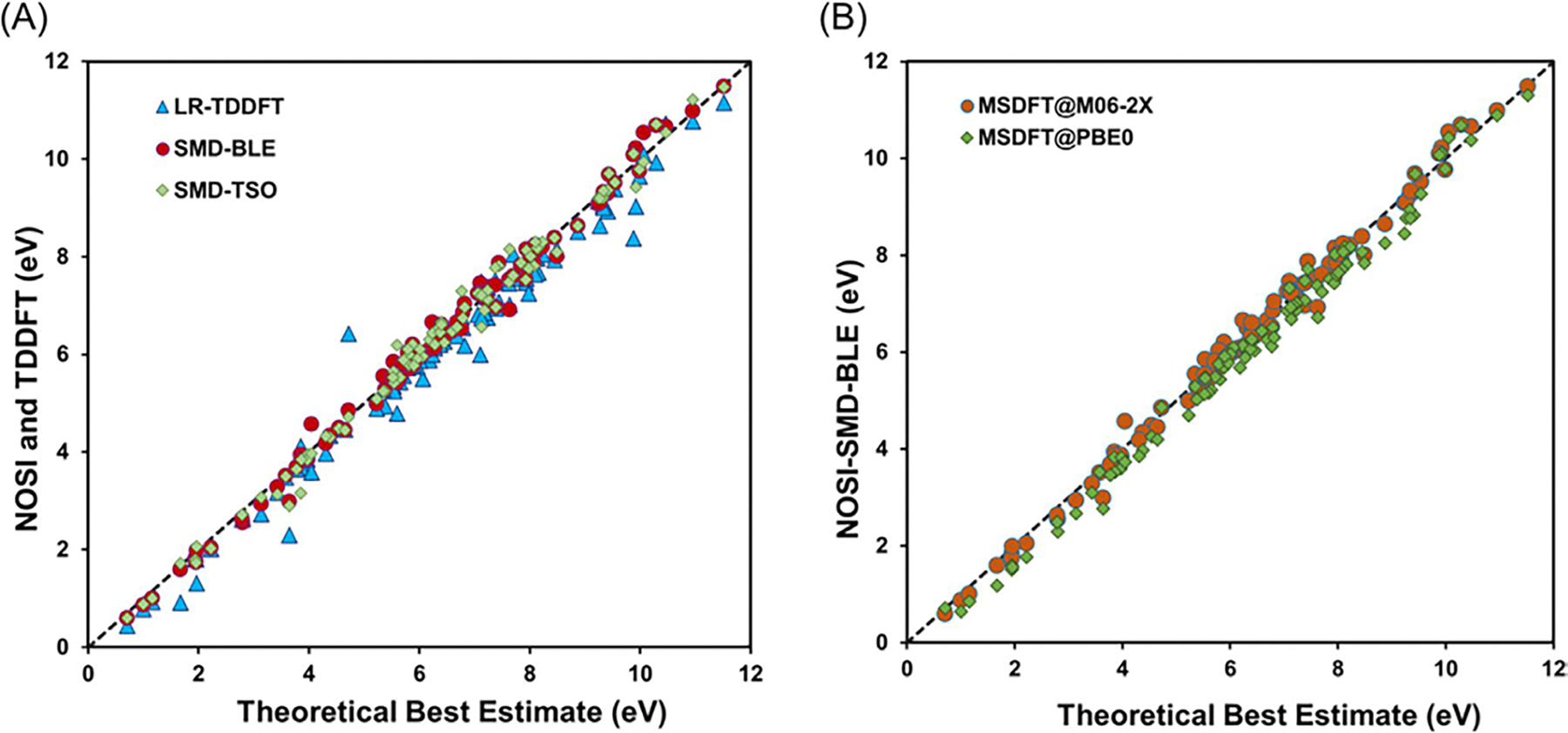
(A) 100 excitation energies of singlet and triplet states from multistate density functional theory (MSDFT) with nonorthogonal state interaction (NOSI) and from linear-response time-dependent density functional theory (LR-TDDFT) against the theoretical best estimates of the Loos2018 dataset [122]. Results from BLE and target-state optimization (TSO) are adopted in NOSI calculations. (B) Comparison of NOSI excitation energies obtained using the meta-GGA M06-2X functional (MSDFT@M06-2X) and the hybrid PBE0 alternative (MSDFT@PBE0) against the same dataset. The M06-2X functional and aug-cc-pVTZ basis functions are used. Reproduced from reference [126] with permission by the American Chemical Society.

Figure 4A, drawn using the data reported by Zhu et al., displays 100 vertical excitation energies for these molecules as determined by both NOSI and LR-TDDFT calculations, plotted against the TBE dataset from Loos et al. [122, 126] The overall agreement between the MSDFT results and the TBE values is excellent, particularly when applying the spin-multiplet degeneracy (SMD) constraint to account for spin-coupling interactions (Equation 49). Using BLE or TSO optimization, the RMSD errors are 0.22 eV and 0.25 eV, respectively, relative to the TBE. Furthermore, both models exhibit minimal systematic errors, with mean-signed errors (MSE) of approximately −0.02 eV and −0.01 eV, respectively. In contrast, for this set of small molecules, LR-TDDFT using the same M06-2X functional shows noticeably larger errors, with an RMSD of 0.42 eV compared to the TBE data.

The dependence of NOSI results on the particular density functional approximation adopted is displayed in Figure 4B. For this purpose, we compare the computed MSDFT excitation energies with the meta-GGA M06-2X functional [124] and the PBE0 hybrid functional alternative [127, 128], denoted as MSDFT@M06-2X and MSDFT@PBE0. In both cases, the BLE optimization method along with the SMD approximation for the transition density functional was used. Figure 4B shows that MSDFT@PBE0 systematically underestimates excitation energies with an MSE of −0.26 eV, similar to that of LR-TDDFT (−0.24 eV) in Figure 4A, although the RMSD error is lightly smaller with MSDFT@PBE0 (0.31 eV) than that from LR-TDDFT (0.42 eV). Back to Figure 4A, one notable error in LR-TDDDFT is the $n^{2}\to {\left(\pi^{*}\right)}^{2}$ double excitation of nitrosomethane, which has a value of 6.42 eV, compared to the TBE of 4.72 eV. NOSI with M062-X and PBE0 functionals yields 4.85 and 4.86 eV, respectively.

In Table 2, Zhu et al. further scutinized the MSDFT-NOSI approximation employing a standard KS-functional developed for the ground state by comparison with a range of 12 popular WFT methods reported by Loos et al. [122]. The coupled-cluster with triple excitations (CCSDT, CCSDTQ and CC3) have the best performance, whereas ADC(2) and ADC(3) as well as CC2 exhibit larger deviations (Table 2). NOSI, coupled with the M06-2X functional developed for KS-DFT, yields results lying between the most accurate CC series that include triples and those second-order CC theories (Table 2). Moreover, we found no notable difference in accuracy for singlet and triplet states from NOSI. For wavefunction theories, the low-cost CCS(D), ADC(2) and CC2, and so forth, have greater MAE in Rydberg states by as much as 0.15 eV than valence excited states. Note that there are 57 valence excited states and 43 Rydberg states displayed in Figure 4A, of which 43 are triplet states. It turns out that NOSI(SMD) performs slightly better for the Rydberg states (MAE: 0.15 eV; RMSD: 0.19 eV) than valence excitations (MAE: 0.18 eV; RMSD: 0.24 eV). This trend follows that found in ADC(3) (MAE of 0.17 eV and 0.28 eV, respectively). The main source of errors in valence excitations in the NOSI method comes from $\pi \to \pi^{*}$ excitations.

Two conclusions can be drawn from the study by Zhu et al. [126]: (i) the performance of MSDFT-NOSI is statistically better than LR-TDDFT using the same KS density functional approximation on 100 vertical excitations of small molecules in the Loos2018 dataset; and (ii) the results using MSDFT-NOSI are dependent on the functional used to optimize individual determinants in the MAS.

**FIGURE 5 |:**
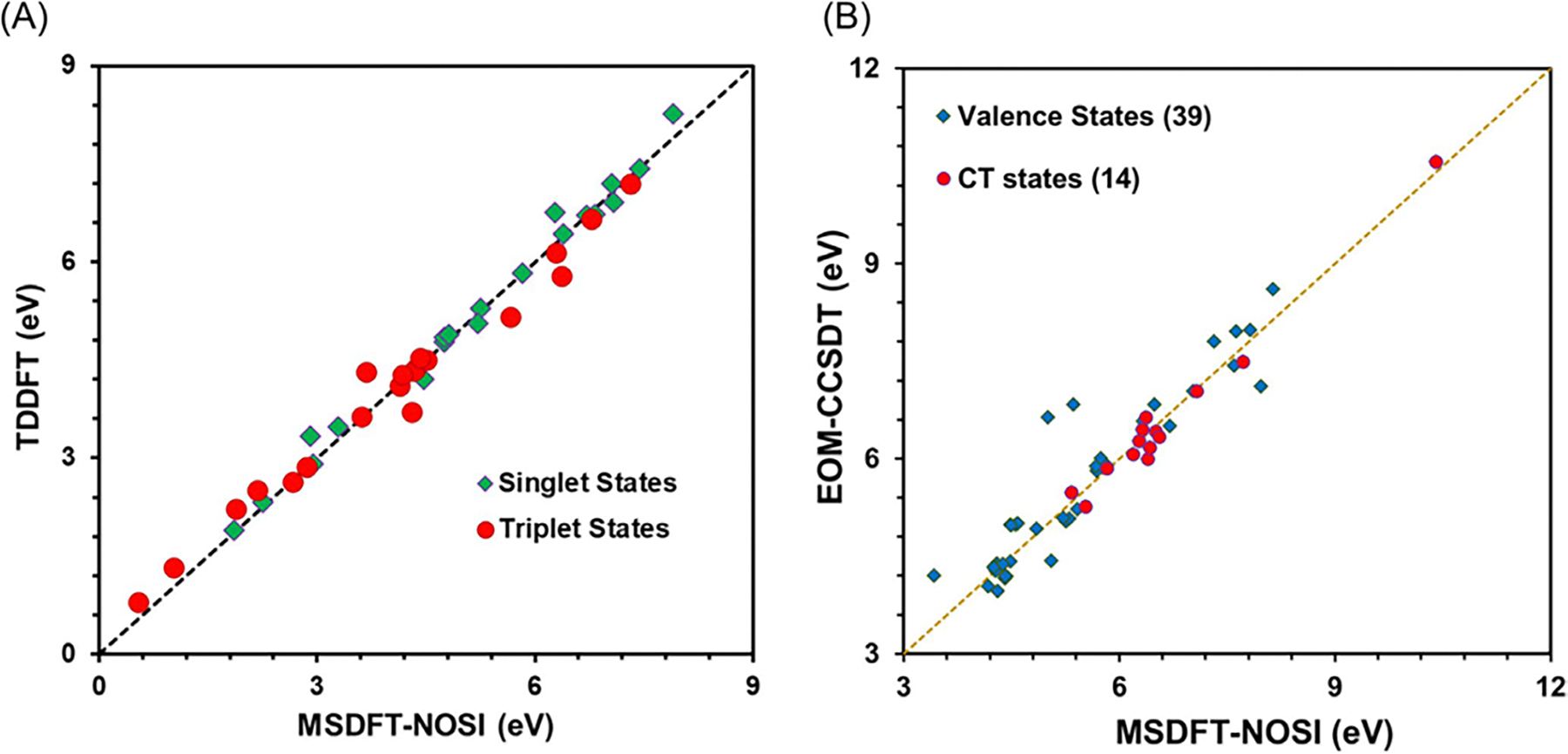
(A) Comparison of singlet and triplet excitation energies of acetone, cis- and trans-dimethoxymethane, methyl vinyl ether, para-chloromethyl anisole, naphthalene, and pentacene determined using MSDFT-NOSI and LR-TDDFT results with M06-2X/cc-pVDZ. Adapted from Reference [123] with permission by the American Chemical Society. (B) Comparison between NOSI@M06-2X and EOM-CCSDT results on local valence (blue) and inter-molecular charge transfer (red) excitations of bimolecular complexes. The cc-pVDZ basis functions were used in both calculations, and data are from References [117, 129].

In energy decomposition analysis of intermolecular interactions for bimolecular complexes in excited states, Hettich et al. [123] reported the first two singlet excited states and two triplet states determined using NOSI@M06-2X/cc-pVDZ with the ground-state geometries of acetone, cis- and trans-dimethoxymethane, methyl vinyl ether, para-chloromethyl anisole, naphthalene, pentacene, and tetracyanoethene, plus two $S_{1}$ excited state geometries of acetone and pentacene. For these representative medium-sized organic molecules, the excitation energies from LR-TDDFT are typically excellent in comparison with experiments. This group of 40 (20 singlet and 20 triplet) excited states extends the size of the above small molecules, and the accord between NOSI and LR-TDDFT results in Figure 5A is also reasonable at an RMSD of 0.25 eV and consistent with their comparisons with the TBE results in the small-molecule set.

#### 6.1.3 | Local and Charge-Transfer Excitations of Bimolecular Complexes

Charge transfer excitations are notoriously difficult for LR-TDDFT, although different approaches have been developed such as range-separated functionals, some adaptively fitted to individual molecules with limited success. Zhao et al. investigated bimolecular complexes between the electron acceptor tetracyanoethene (TCNE) and a series of aryl compounds, including benzene, toluene, o-xylene, naphthalene and anthracene using NOSI with the M06-2X, PBE0, and B3LYP functionals along with the cc-pVDZ and cc-pVTZ basis sets [117]. In these MSDFT-NOSI calculations, non-aufbau occupation-constrained orbitals are optimized using the BLE method, in which Kohn–Sham orbitals are strictly block-localized by expanding only over basis functions located on individual monomers. Therefore, charge-transfer states are constructed by block-localization as diabatic states. It turns out that NOSI calculations yield similar results with the three density functional approximations, but the larger basis set tends to underestimate CT excitation energies by 0.1–0.2 eV [117]. Standard Kohn–Sham functionals severely underestimate the excitation energies of inter-fragment CT states using LR-TDDFT. For the range-separated BNL functional, the default parameter surprisingly overestimates the first CT excitation energies, but specific optimization of separation parameter for each complex results in a good agreement with experiments (±0.2 eV) [130]. The RMSD error between MSDFT@M06-2X/cc-pVDZ results and experiments is only 0.12 eV for the seven experimentally resolvable CT states (Table 3). The ionization potential-fitted CAM-QTP-02 functional with the cc-pVTZ basis set is also reasonable with an RMSD of 0.34 eV for these compounds.

Szalay and coworkers investigated local valence excitations and CT excited states on a set of two-component complexes, including H_3_N⋯F_2_, acetone⋯F_2_, pyrazine⋯F_2_, H_3_N⋯OF_2_, acetone⋯nitromethane, pyrazine⋯NH_3_, pyrrole⋯pyrazine, stacked pyrrole⋯pyrazine, and tetrafluoroethene⋯ethene [129, 131, 132]. Equation-of-motion coupled cluster with singles, doubles and triples (EOM-CCSDT) and iterative EOM-CCSDT-3 were used along with a family of “low-cost” models such as EOM-CCSD, CC2 and CC3, and ADC(2). Although strictly local excitation was not possible in delocalized calculations in that work, MSDFT employing block-localized excited configurations in the MAS ensures the local (valence) excitation within one molecular fragment in the presence of the other monomer in its ground state. Figure 5B exhibits 39 local valence excitations and 14 CT excited states as compared with the results from EOM-CCSDT calculations, both using cc-pVDZ basis functions. The overall RMSD error is 0.48 eV between the two methods; Figure 5B clearly show several outliers in the correlation plot, notably from a systematic underestimation of three local excitations of OF_2_, without which the RMSD is reduced to 0.29 eV.

EOM-CCSDT treats charge-transfer state properly as seen in convergent results from different methods in the work of Kozma et al. [129] In MSDFT-NOSI, Zhao et al. introduced a MAS consisting of singly excited configurations of key frontier orbitals plus forward and backward CT configurations. It was found that good accord was obtained for an RMSD of 0.20 eV over the 14 interfragmental CT excited states between NOSI calculations employing the Minnesota M06-2X functional approximation and EOM-CCSDT results [117]. It was noted that the computational cost of the MSDFT-NOSI method, unlike the exponential scaling with the size of the system in multiconfigurational wavefunction methods, is comparable to M separate KS-DFT optimizations, where M is the number of determinants in the MAS. Importantly, these calculations can be performed in parallel independently.

**FIGURE 6 |:**
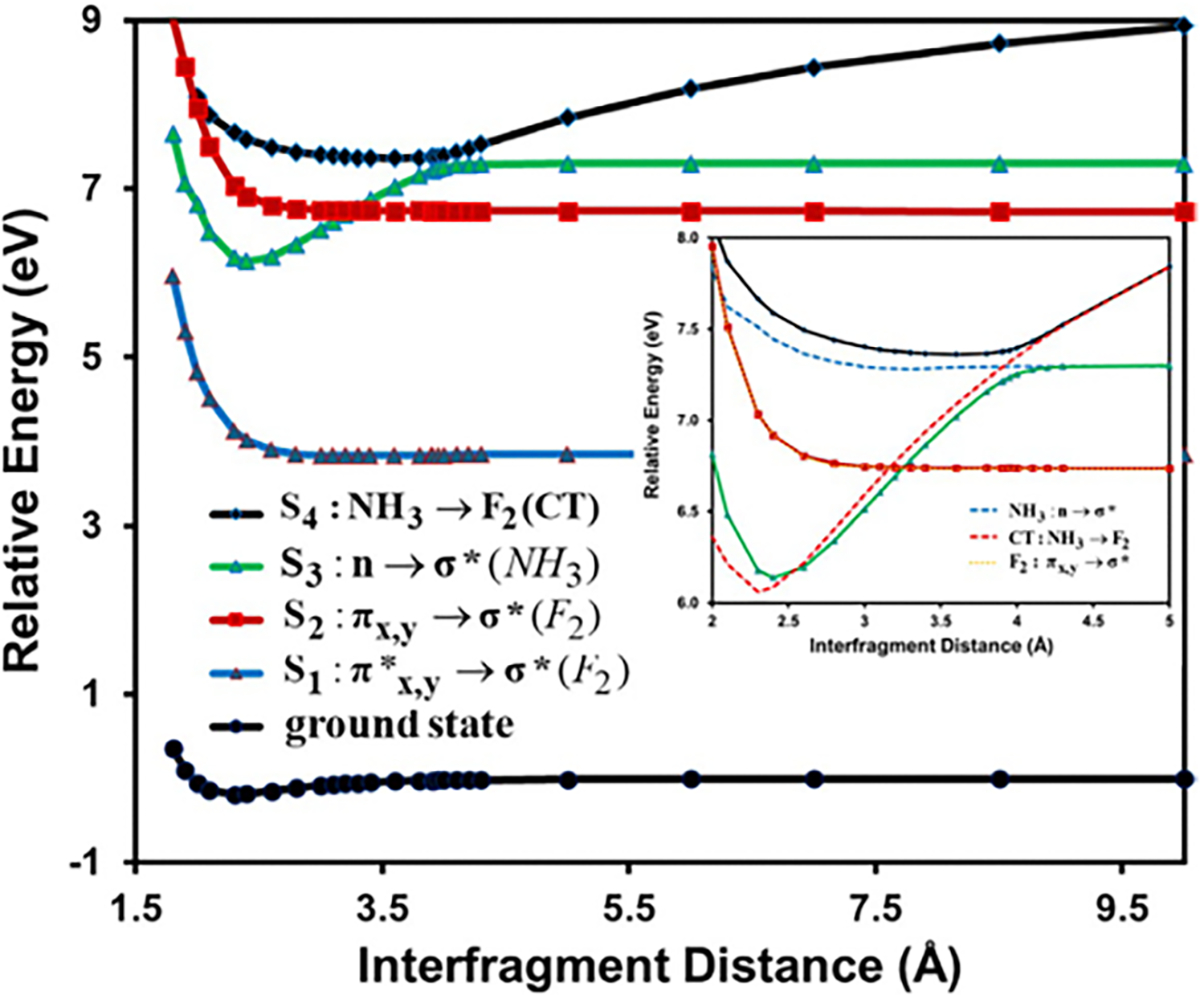
Potential energy curves of the adiabatic ground state, the first three singlet valence excited states and the NH_3_ to F_2_ charge transfer state as a function of N-F distance (angstroms) of the NH_3_⋯F_2_ complex. All calculations are performed using MSDFT-NOSI with the M06-2X functional and cc-pVDZ basis set and energies are given relative to the fully separated monomers. Insert shows the diabatic states (dotted curves) for the _3_ → F_2_ charge-transfer, and local excitations of $n\to \sigma^{*}$ of NH_3_ and $n\to \pi^{*}$ of F_2_, determined by using the generalized diabatic at construction (GDAC) method. Figure is based on data from Reference [117].

In closing this section, we highlight the computation of potential energy curves of the lowest four excited states, including both local valence and intermolecular charge-transfer states, in the H_3_⋯F_2_ complex in Figure 6. Clear features of a CT state corresponding to an electron transfer from NH_3_ to F_2_ can be observed by its strong dependence on inter-fragment separation [117]. The delicate features of avoided crossing with the doubly degenerate $\pi_{x,y}\to \sigma^{*}$ states of F_2_ and an avoided crossing with a local $n\to \sigma^{*}$ excitation of ammonia can be further analyzed by examining the specific diabatic states using the generalized diabatic-at-construction (GDAC) method (inset of Figure 6).

### 6.2 | Core-Level Excitation

The block-localization scheme of optimizing excited configurations, using either the BLE or the TSO method, is useful for another class of applications, namely the calculation of core-level excited sates. These applications can be used to study X-ray spectra such as near-edge X-ray absorption spectroscopy (NEXAS), X-ray photoelectron spectroscopy (XPS), and X-ray photoelectron spectroscopy (XPS). Because the coupling between core and valence excited states can be fully ignored, or core-valence separation (CVS) [133], a MAS that includes only the relevant core to valence excitations can be constructed. In these applications, full orbital relaxation, especially including those for core electrons, is critical to determining quantitatively the energies of core-level excited states, without which the absolute errors can be enormous using LR-TDDFT. Therefore, the closely related, state-specific ΔSCF-DFT procedures have been widely used and successful after spin-projection correction to the spin-mixed determinant states [134]. Concerning spin-projection correction to the spin-mixed configuration $E_{\mathrm{mix}}\left(\Xi^{↑↓}\right)$, while it is straightforward for the singlet-triplet pair with $2E\left[\Xi^{↑↓}\right]-E_{t}\left[\Xi^{↑↑}\right]$, there is no simple formalism involving higher spin states. In MSDFT-NOSI, the spin-multiplet degeneracy (SMD) method can be generalized to any spin coupling interactions [117].

A major problem with direct ΔSCF-DFT, state-specific calculations is that it is not certain if an eigenstate corresponding to the desired excited state is in fact obtained in order to compare with experimental findings. For excitations from the 1 s orbital of carbon, nitrogen and oxygen to low-lying valence orbitals of closed-shell molecules and to the singly occupied molecular orbital of free radical species, there is little mixing with the other states and the excitation energies after spin-projection are generally very good. However, for excitations to unoccupied orbitals of radicals, strong coupling among spin-complementary configurations and with other doublet states exist. In this case, such single determinant optimizations are inadequate. On the other hand, this is not an issue in MSDFT-NOSI calculations since state interaction is incorporated in the diagonalization of the Hamiltonian matrix functional. In NOSI, BLE and TSO optimized excited determinants are used as the basis states in a MAS.

**FIGURE 7 |:**
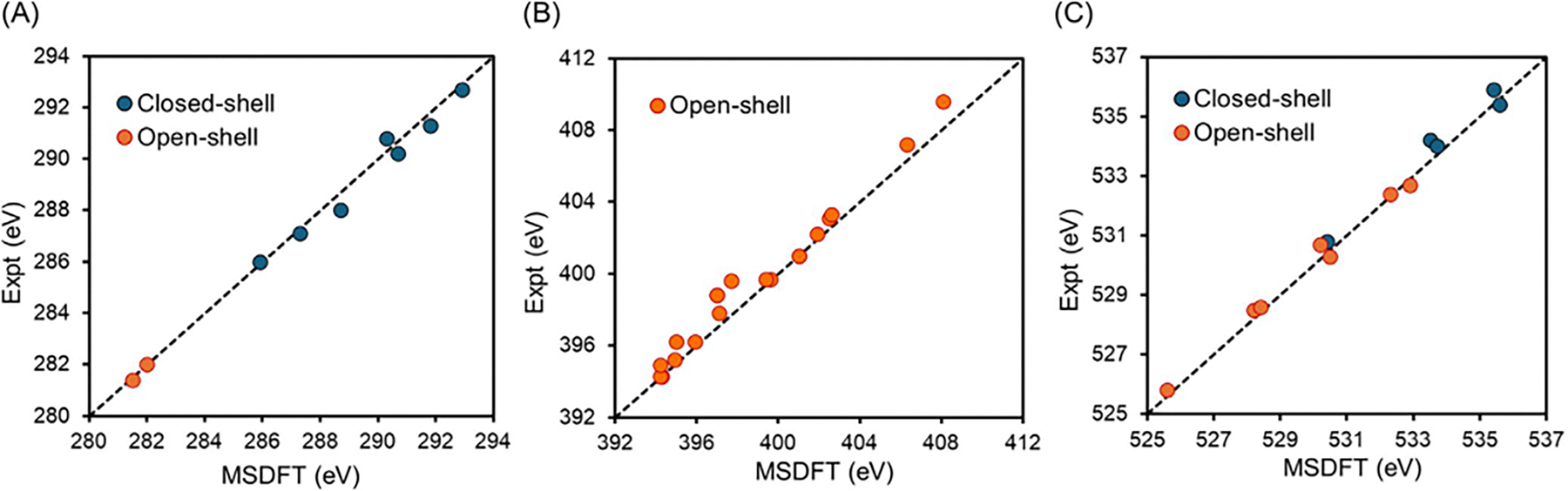
Computed and experimental K-edge excitation energies (eV) from the 1s orbital of (A) carbon atom, (B) nitrogen atom, and (C) oxygen atom of closed-shell (blue) and open-shell (brown) molecules and ions. The block-localized excitation (BLE) method is used in MSDFT calculations of open-shell radicals and ions with the BLYP functional and def2-QZVP basis functions, whereas the target-state optimization (TSO) is adopted for closed-shell compounds. In TSO calculations, the hybrid B3LYP functional is used along with cc-pVTZ basis functions. Figures are drawn using data reported in reference [117] for open-shell systems and from reference [111] for closed-shell molecules.

The TSO optimization technique was used to determine core-to-valence excitation energies and ionization potentials for a set of small organic molecules, including CH_4_, CH_4_OH, CH_3_CN, H_2_C=O, H_2_O and CO_2_ [111]. In these calculations, the specific 1 s Kohn–Sham orbital optimized in KS-DFT calculation for the ground state is extracted and placed in an isolated block, separated from the remaining orbitals. Although the isolated 1 s orbital is frozen and unoccupied, effectively creating a hole orbital, the remaining orbitals, reconstructed using the rest of the KS-orbitals as basis, are fully relaxed and optimized. First, it was found that the RMSD error for the computed ionization energies of 1 s electrons of carbon and oxygen atoms is just 0.38 eV relative to experimental values using a standard hybrid B3LYP functional and the cc-pVTZ basis set. For carbon and oxygen core-to-valence excitations, including 3 s, 3p and $\pi^{*}$ states, the optimization after spin-projection correction yielded an RMSD of 0.44 eV in excitation energies for 12 states in comparison with experiments (Figure 7).

MSDFT-NOSI employing the BLE optimization was applied to core-level excitations of free radical species using BLYP and M06-2X density functional approximations with the def2-QZVP and aug-cc-pCVQZ basis sets [117]. Relativistic effects were considered and found to have contributions of 0.1, 0.2, and 0.4 eV for carbon, nitrogen, and oxygen atoms. Because they are relatively small, relativistic effects are not corrected in the reported data [117]. For core 1 s electron excitation into the singly occupied Kohn–Sham orbital of free radicals, the study showed that the BLYP functional performs slightly better than the meta-GGA alternative with RMSD errors of 0.18 and 0.67 eV, respectively [117]. The M06-2X functional seems to systematically underestimate core excitations by about 0.3 eV for this small sample of radicals. In that study, the spin-adapted TDDFT, denoted as X-TDA, which includes a correction by random phase approximation, was also used, but the half-and-half hybrid BH&HLYP functional was used to reduce the effect equivalent to charge transfer [135]. Still, the deviation in computed excitation energies was much larger at an RMSD of 3.8 eV.

For the multiconfigurational doublet states, there is no apparent difference in performance between BLYP and M06-2X in MSDFT calculations. Of the 14 multiconfigurational states in the nitrogen-containing radicals, three states from NH^+^ have errors close to 2 eV. If these three states are excluded, the root-mean-square errors from MSDFT calculations using M06-2X and BLYP functionals are, respectively, 0.5 and 0.6 eV. Note that the errors in core-excited states are typically more than 10 eV from LR-TDDFT calculations on closed-shell molecules, which cannot be directly applied to these open-shell systems since states involving two-electron excitations are needed. The X-TDA method includes spin-adapted configurations, as does in MSDFT, the standard error is 4.1 eV using the B&HLYP functional [117].

Another interesting example is the core excitations of the benzene cation radical, first reported in a joint experimental and computational study. Table 4 summarizes the computed excitation energies from the MOs of the six core orbitals using MSDFT@M06-2x and X-TDA, both using the aug-cc-pVTZ basis set, along with experimental data and computational results by using EOM-CCSD/6-311(2+)G**(uC) basis set [117, 136, 137]. The latter values have been systematically shifted by 0.75 eV to match experiments. The dominant experimental spectral peaks (E, F, G and H) are easily matched by all methods in good agreement. Of note is that the absolute excitation energies from MSDFT calculations do not need to be shifted as in other cases. Furthermore, it was suggested that many computed excitation energies can be assigned to weaker peaks, clearly identifiable, but they have not been discussed nor assigned in the experimental study. Both EOM-CCSD and MSDFT calculations produce the same group of excitation energies in the reported spectral region.

Overall, these studies show that MSDFT employing a MAS can be used to model core excitations. Since orbitals are fully relaxed in response to core electron excitation, the computed absolute excitation energies are in good accord with experiments. Not only can MSDFT-NOSI be applied to closed-shell molecules, it is also effective in studying open-shell systems. Although the method is not a black-box approach—because one needs to first define a MAS for specific applications—the configurations can provide an intuitive interpretation of the electronic transitions in diabatic states.

### 6.3 | Excited-State Processes

#### 6.3.1 | Balancing Static and Dynamic Correlation in Photo-Dissociation of LiF

**FIGURE 8 |:**
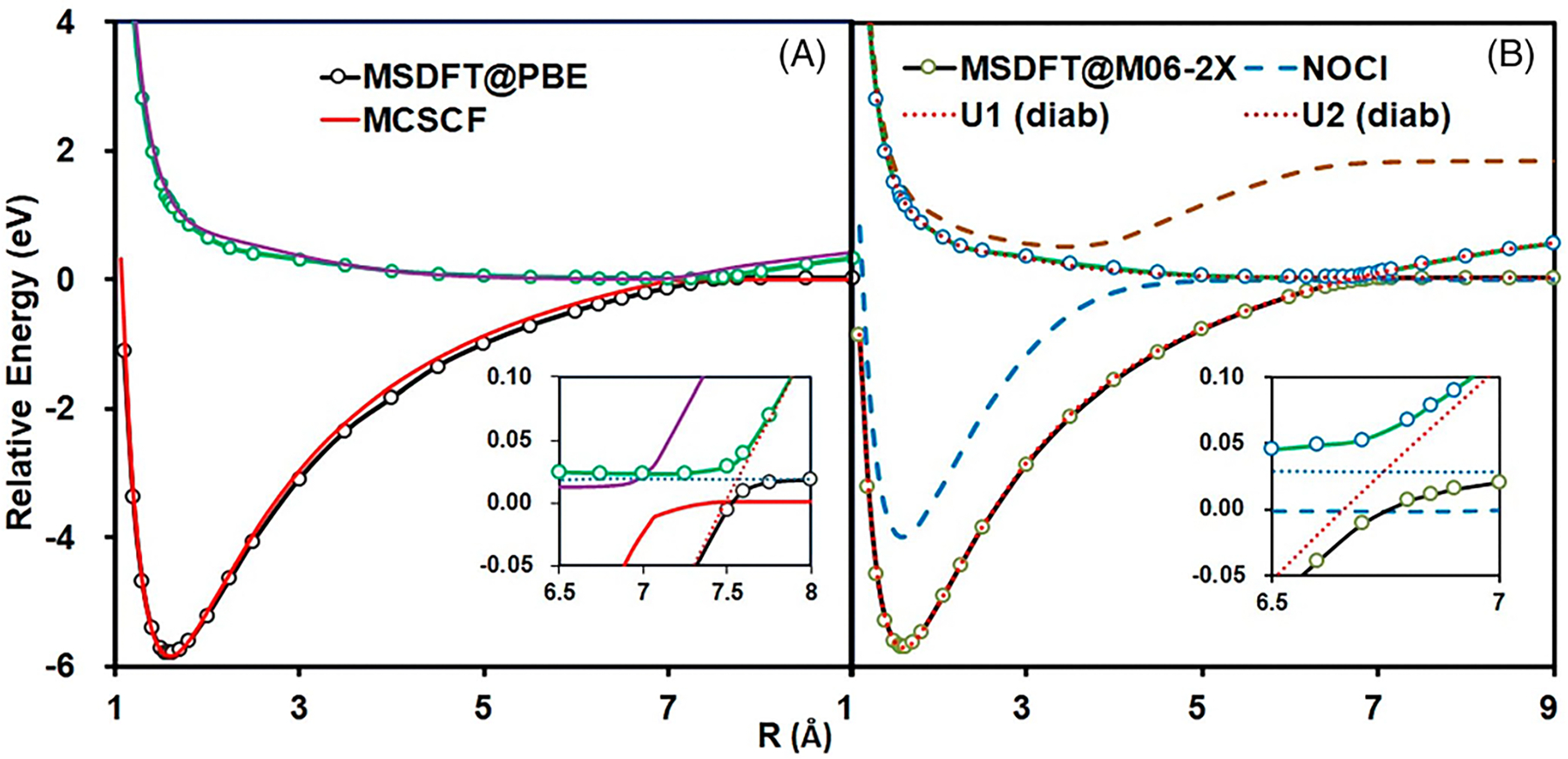
Adiabatic (solid) and diabatic (dotted) potential energy curves depicting the avoided crossing of the Σ+1 states of LiF along the interatomic distance, determined using MSDFT with the PBE functional (MSDFT@PBE) in (A) and with the MS06-2X function (MSDFT@M06-2X) in (B). The MCSCF results from [138] are shown in red in (A) and the NOCI potential energy curves are given in (B) for comparison. The aug-cc-pVQZ basis was used. In MSDFT-NOSI calculations, atom-block localized orbitals are used to represent the ionic and covalent states of LiF. In particular, the covalent and ionic states are defined by assigning 2, 3, and 4 electrons to the Li block and the remaining electrons to the F fragment, corresponding to the valence bond-like configurations of Li ^+^F^−^, Li*F*, and Li^−^F^+^. To account for charge delocalization, the $2s\to 2p_{z}$ excited state of lithium, and two π covalent states are included; here, the basis functions of Li are further separated into two blocks, one corresponding to those of the $p_{x,y,z}$ symmetry and one for the rest of the Li atomic orbitals. Each of the 4 covalent states is represented by two Slater determinants from the ${Li}^{*}(↑)F^{*}(↓)$ and ${Li}^{*}(↓)F^{*}(↑)$ combination. Adapted from [17] with permission by the American Chemical Society.

The photo-dissociation of LiF is a classic example that shows delicate balance of dynamic and static correlation that results in an avoided curve crossing at an extraordinarily long interatomic distance (ca. 7 Å) and a very small energy gap between the ground state and the $S_{1}$ excited state. Early on, Werner et al. established the potential energy curves at a quality of FCI, which has served as a standard to test computational methods. Shown in Figure 8 are the computed potential energy curves along the diatomic Li-F bond length, using MSDFT-NOSI with the PBE and M06-2X functionals along with the multiconfigurational wavefunction results [138–140].

Figure 8 shows that both PBE and M06-2X in NOSI calculations resulted in an adequate description of the key features of the potential energy curves, with the avoided crossing taking place at 6.75 Å from M06-2X and 7.55 Å from PBE along with a small energy gap of 0.06 and 0.03 eV by the two density functionals. Importantly, although the character of the ground state changes rapidly from a predominantly ionic configuration to the covalent state, the transition occurs smoothly in the computed curves. For comparison, the Werner study showed an curve-crossing distance at 7.05 Å (ΔE=0.05eV) and an experimental estimate of about 7.3 Å (ΔE=0.02eV) [138]. Without dynamic correlation in nonorthogonal configuration interaction (NOCI) calculation, using either the MSDFT orbitals (Figure 8b) or optimized orbitals, and in CASSCF calculations without dynamic correlation correction, the qualitative trends cannot be duplicated, having much shorter distances (ca. 4–5 Å) and huge energy gaps (ca. 1 eV) [86]. Worse still, applying the second-order perturbative correction (state-specific CASPT2) to each potential energy curve obtained by CASSCF results in “double crossings” for lacking basic state interactions [58]. Of course, the poor qualitative behavior can be rescued by including state interaction in the perturbative treatment, such as the XMS-CASPT2 approach [141–143].

#### 6.3.2 | Conical Intersection

##### 6.3.2.1 | Symmetry-Induced Conical Intersection.

Jahn-Teller active molecules, such as cyclopropenyl radical $\left(C_{3}H_{3}^{•}\right)$, represent a special type of conical intersection, where the degeneracy of the electronic state is lifted by symmetry-broken vibrations. Following an insightful analysis Paterson et al. [144], the Jahn–Teller geometries at the conical intersections are optimized for cyclopropenyl radical at the $D_{3h}$ symmetry. In this case, effective valence bond orbital blocks were used to define the three possible spin-localized configurations.

**FIGURE 9 |:**
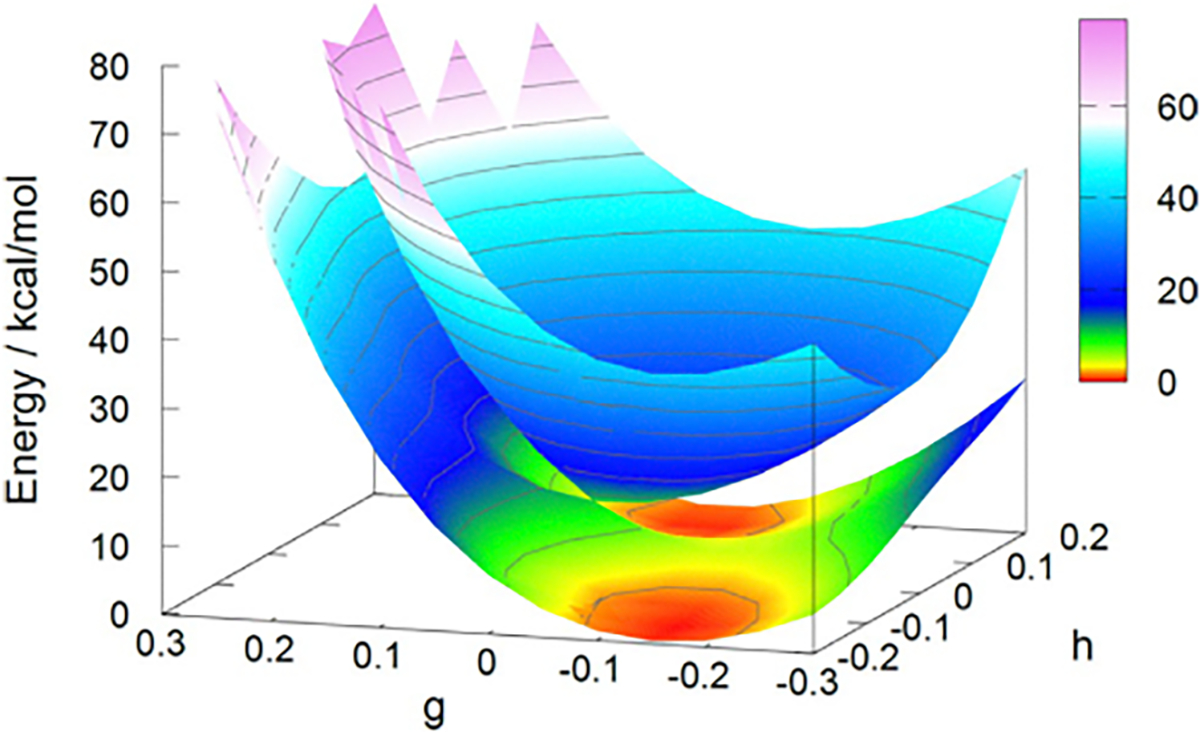
Computed potential energy surfaces for the ground and the first excited states for cyclopropenyl radical along the gradient difference (g) and derivative coupling (h) vector directions. Adapted from [62] with permission by the American Chemical Society.

In each configuration, four orbital blocks were partitioned, representing the σ-framework (consisting of 18 electrons, 5 from each carbon plus 3 from the three hydrogen atoms) and three singly occupied p-orbitals on carbon atoms [62]. At the symmetry-induced conical interaction, the gradient difference (GD) and derivative coupling (DC) vectors correspond to the $e^{\prime }$ normal vibrational modes [144], along which the molecular structure is stretched and compressed to produce potential energy surfaces for the doublet ground and first excited state. Figure 9 shows that distortion along the GD(g) vector leads to a saddle point of $C_{2v}$ symmetry on the ground-state PES, which has one short and two long CC bonds. The two minima also have C2v symmetry but one long and two short CC lengths. Moving away from the $C_{2v}$ symmetry along the DC(h) direction results in a cone from MSDFT calculations. LR-TDDFT produces a spurious seam with KS-DFT energy higher than that of the TDDFT excited state. Of course, the topology of conical intersection is not formed from LR-TDDFT calculation.

##### 6.3.2.2 | Photodissociation of Ammonia.

The photochemical process, ${NH}_{3}\;\to \;{NH}_{2}\;\cdot \;+\;\text{H}\cdot$, is a classic example that have been extensively studied [145, 146]. At the crossing point between the NH2B21 and NH2A21 states along the bond dissociation coordinate in the molecular plane, bending about the molecular plane lifts the energy degeneracy, resulting in a conical intersection with a double cone feature in the two potential energy surfaces for the ground state and the first excited state. Previously, we have used NOSI to determine the potential energy curves along the N−H stretch coordinate, employing a set of 12 valence bond configurations [62]. Here, to illustrate the MS-SCF method in MSDFT calculations, we reconstructed the potential energy surfaces using delocalized molecular orbitals in an active space consisting of 4 electrons and three orbitals, rather than the localized valence bond states previously [82]. Since we have not fully implemented the nonorthogonal optimization algorithm, the present calculations are performed employing a common set of orthogonal orbitals, which are adequate in view of the small size of the orbital space. We found that inclusion of single and double excitations for a total of five spin-adapted configurations are sufficient, and this example shows that MS-SCF can be conveniently adapted into any code with MC-SCF (CASSCF) capability.

**FIGURE 10 |:**
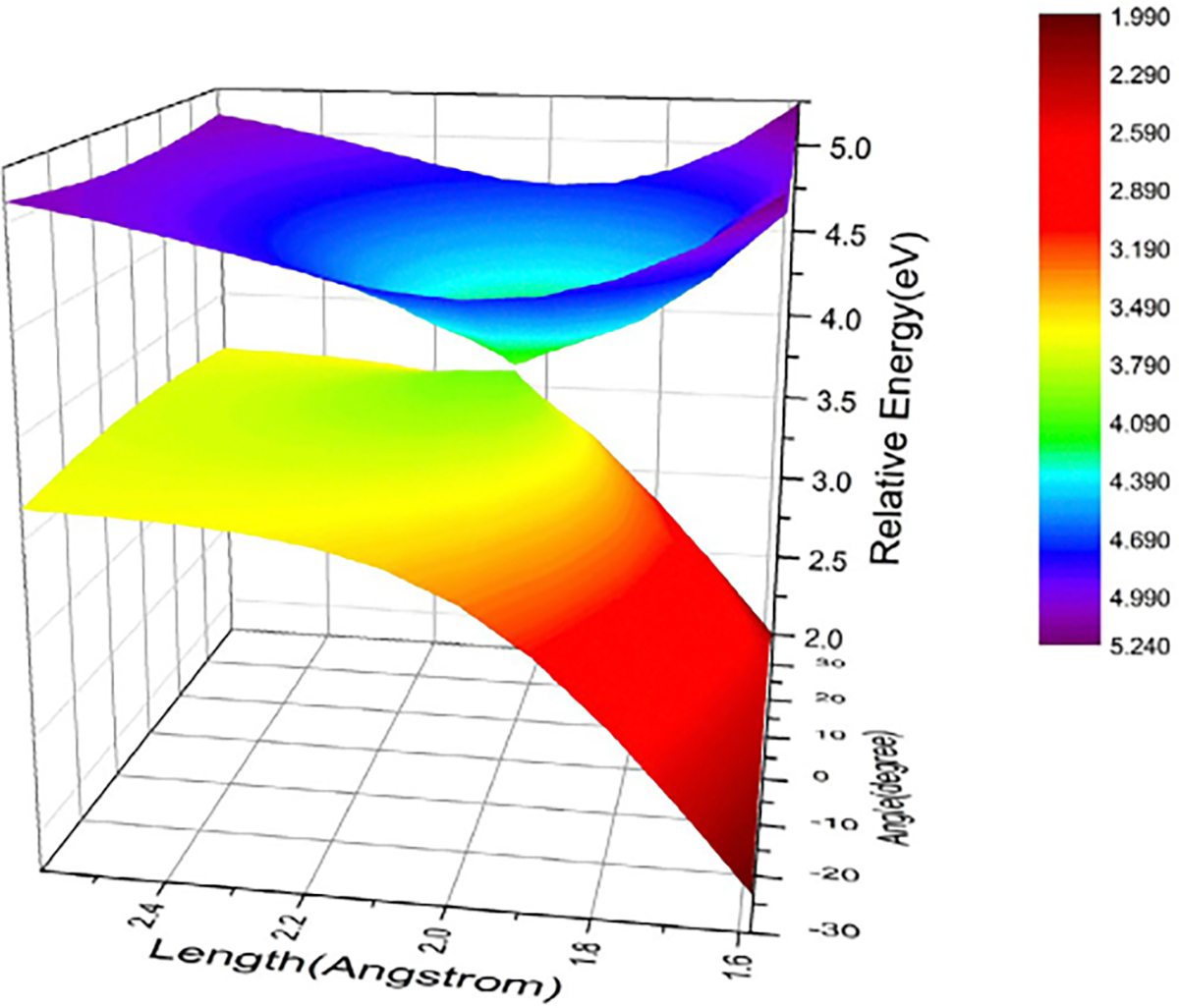
Potential energy surfaces of the ground and first excited states as a function of the N−H bond-dissociation coordinate (in Å) and its bending angle about the plane of the NH_2_ group (in degrees). The PBE approximate functional is used with the cc-pVTZ basis set. An active space consisting of four electrons and three orbitals is used with single and double excitations in multistate self-consistent-field (MS-SCF) optimizations. Energies are given in eV. Adapted from [82] with permission by the American Chemical Society.

Figure 10 displays the potential energy surfaces for the ground and first excited states in the bond-dissociation coordinate along one of the N−H bonds and its bending angle from the plane of the NH_2_ group. The conical intersection point occurs at a distance of 2.001 Å with an energy of 5.00 eV above that of ammonia, which may be compared with values of 1.990 Å and 5.16 eV in the full-dimension potential energy surface [145]. The topology of the PES about the conical intersection point is correctly produced from MS-SCF optimizations [82].

### 6.4 | Diradicals

#### 6.4.1 | Singlet-Triplet Energy Gap

##### 6.4.1.1 | Cyclobutadiene.

**FIGURE 11 |:**
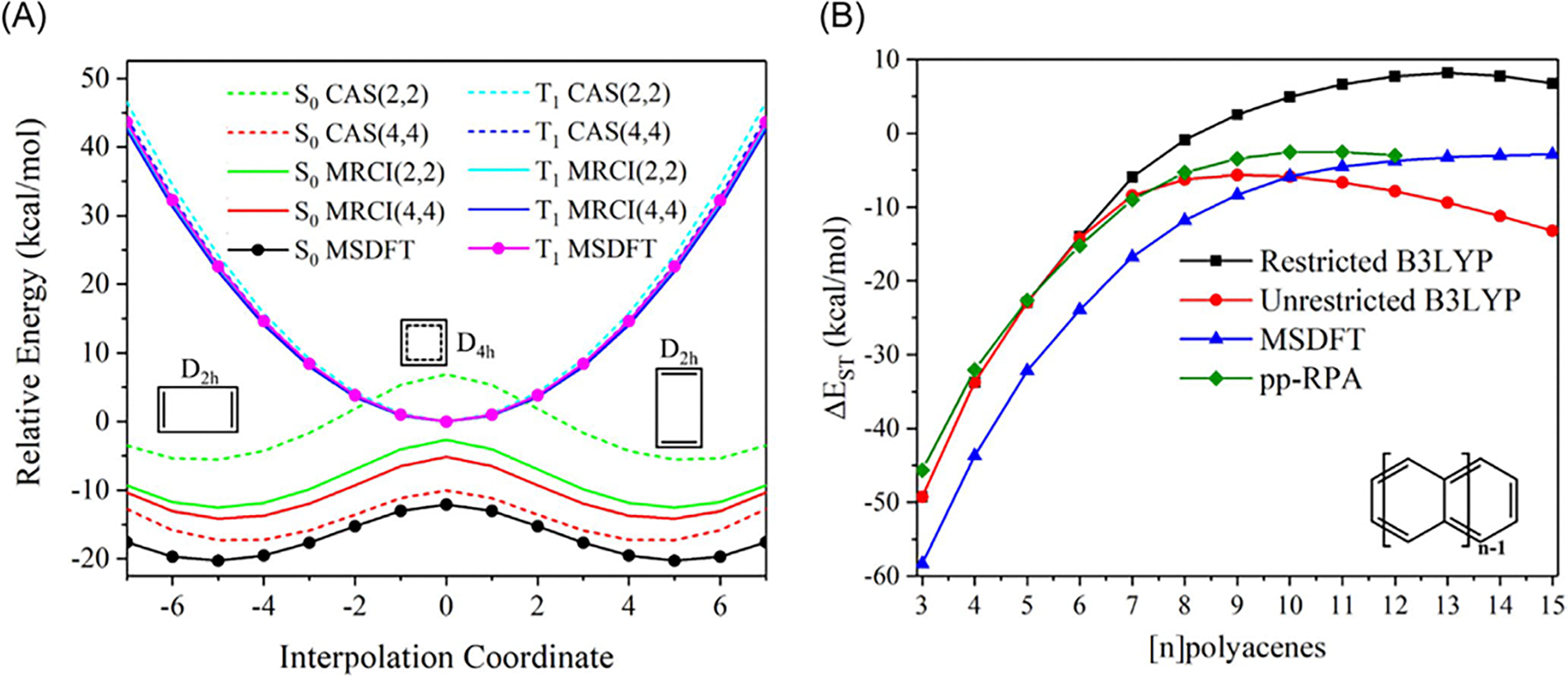
(A) Computed potential energy curves for the ground-state singlet and triplet states of cyclo-butadiene along the interpolated reaction coordinate between the two rectangular (D2h) geometries at a unitless value of ± 5 through the transition state geometry (D4h). M06-HF functional and cc-pVTZ basis set are used in MSDFT calculations. (B) Computed adiabatic singlet-triplet-energy gap $\Delta E_{ST}$ in kcal/mol for the polyacene series. M06-HF functional and 6–31G(d) basis set are used in restricted (RDFT) and unrestricted (UDFT) DFT, and MSDFT calculations. Adapted from [147] with permission by MDPI, Basel, Switzerland.

The interconversion of two rectangular isomers of cyclobutadiene features a transition state of square $D_{4h}$ geometry that must be treated by multiconfigurational methods. Consequently, KS-DFT is not adequate to describe this process. Interestingly, the transition structure on the singlet ground state corresponds to a minimum on the triplet state. MSDFT-NOSI was used [147], using an active space consisting of two electrons and two orbitals, to model this process and compared with results from CASSCF and CASSCF/MRCI calculations with two-electron-in-two-orbital [2,2] and four-electron-in-four-orbital [4, 4] active space. Of course, MSDFT employs nonorthogonal orbitals independently optimized for different configurations, but a common set of orbitals are used in CASSCF and MRCI. The variations in singlet-triplet energy gap, $\Delta E_{ST}=E_{T}-E_{S}$ from different methods are displayed in Figure 11A along a pathway interpolated between the two isomers.

Interestingly, the potential energy curves in Figure 11A for the triplet state are essentially the same from different methods and, thus, used to anchor the energy plot relative to the minimum of the triplet state energy. Except the minimal CASSCF[2,2], which places the singlet state above the triplet at the square geometry, all other methods yield the correct trends. Inclusion of dynamic correlation using CASSCF[2,2]/MRCI correctly recovers the energy trends, but the energy gap $\Delta E_{ST}$ is too small. Expanding the size of active space, CASSCF [4] produced a ground-state energy curve in close accord with MSDFT and this behavior has been called spin-dynamics in the literature [148]. At the transition structure, MSDFT yields a singlet-triplet gap of 23 kcal/mol, which may be compared with a value of 16 kcal/mol from SF-EOM-CCSD calculation [149].

##### 6.4.1.2 | Polyacenes.

The change of singlet-triplet energy gap in the polyacene series up to n=15 fused benzene rings were determined by using MSDFT with a MAS of two-electrons and two-orbitals, as it should be in the context of efficiency in DFT. Figure 11B shows the computed $\Delta E_{ST}$ from DFT calculations, including restricted and unrestricted KS-DFT, particle–particle random phase approximation (pp-RP A) and MSDFT. There is a large number of computational studies of this system, but we only highlighted a recent study by Yang and coworkers here for comparison [150]. Neither RDFT nor UDFT, using M06-HF and the 6–31G(d) basis set, exhibit the correct trend, where RDFT incorrectly predicted an inverse $\Delta E_{ST}$ and UDFT showed a maximum at 9 fused rings with a quick decrease in $\Delta E_{ST}$ as the number of rings increases (Figure 11B). The pp-RPA shows similar behavior as that of UDFT, although at a slower rate. On the other hand, MSDFT predicted that the energy of the singlet state is uniformly lower than the triplet state in Figure 11B as the size of the linear polyacene increases. This trend is in good accord with the finding from a study employing density matrix renormalization group (DMRG), showing a lower ground-state than the triplet state approaching the infinite chain limit at 2–12 kcal/mol [151].

#### 6.4.2 | Hydrogen-Atom Transfer Reaction

**FIGURE 12 |:**
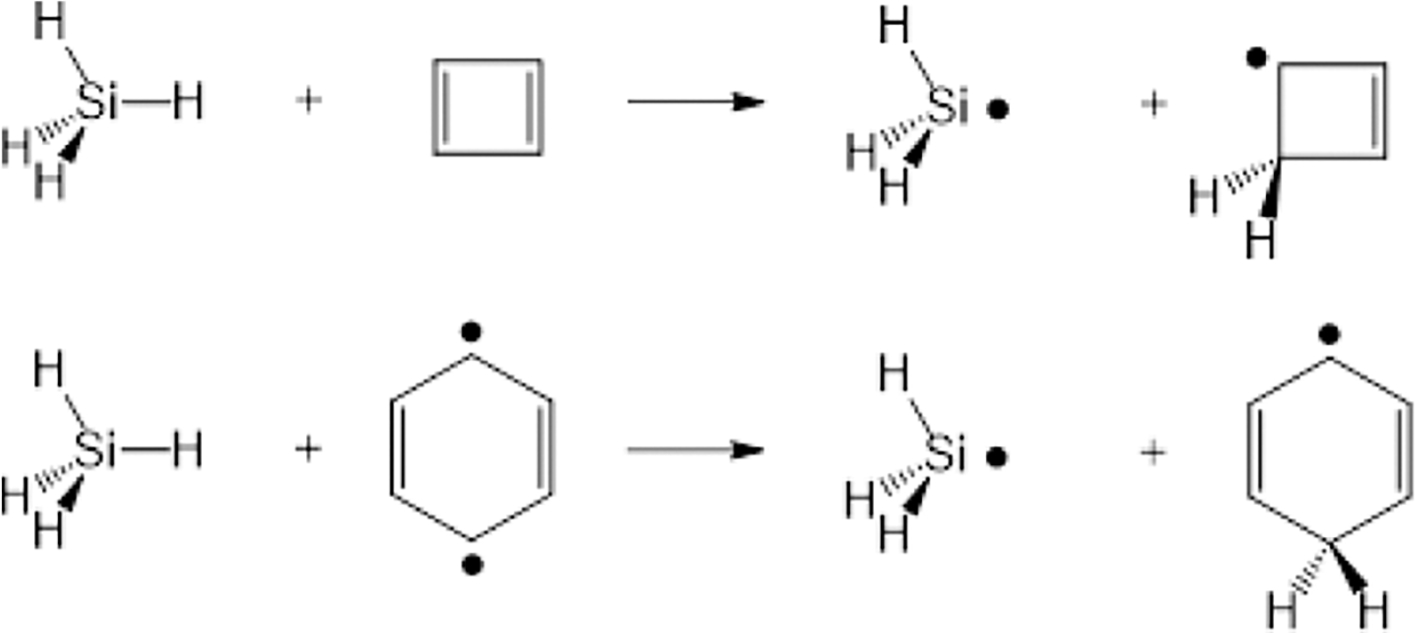
Hydrogen atom transfer reactions of SiH_4_ by the biradical species cyclobutadiene (top) and parabenzyne (bottom).

In the work of Han et al. [147], the hydrogen-atom transfer reactions of silane (SiH_4_) with butadiene—a π-type biradical—and parabenzyne—a σ-type biradical—were examined (Figure 12), with the specific aim of elucidating whether the diradical character during the reaction arises predominantly from two closed-shell configurations or from open-shell radicals. To achieve this, three diabatic states were defined: two representing the reactant state (R) and one corresponding to the hydrogen-atom transfer (HAT) product (P) state:

(52) $$\Theta_{\mathrm{CS}}^{R}=C_{CS}^{20}\overset{ˆ}{A}\left\{\left(\chi_{DR}^{\mathrm{core}}\chi_{HOMO}^{2}\right)\left(\phi_{{SiH}_{4}}^{\mathrm{core}}\phi_{HOMO}^{2}\right)\right\}\;\;+C_{CS}^{02}\overset{ˆ}{A}\left\{\left(\chi_{DR}^{\mathrm{core}}\chi_{LUMO}^{2}\right)\left(\phi_{{SiH}_{4}}^{\mathrm{core}}\phi_{HOMO}^{2}\right)\right\}$$

(53) $$\Theta_{OS}^{R}=\frac{1}{2}\left[\overset{ˆ}{A}\left\{\left(\chi_{DR}^{\mathrm{core}}\chi_{HOMO}^{\alpha }\chi_{LUMO}^{\beta }\right)\left(\phi_{{SiH}_{4}}^{\mathrm{core}}\phi_{HOMO}^{2}\right)\right\}\right.\;\;\left.+\overset{ˆ}{A}\left\{\left(\chi_{DR}^{\mathrm{core}}\chi_{HOMO}^{\beta }\chi_{LUMO}^{\alpha }\right)\left(\phi_{{SiH}_{4}}^{\mathrm{core}}\phi_{HOMO}^{2}\right)\right\}\right]$$

(54) $$\Theta_{HAT}^{P}=\frac{1}{2}\left[\overset{ˆ}{A}\left\{\left(\chi_{DRH}^{\mathrm{core}}\chi_{HOMO}^{2}\chi_{LUMO}^{\alpha }\right)\left(\phi_{{SiH}_{3}}^{\mathrm{core}}\phi_{HOMO}^{\beta }\right)\right\}\right.\;\;\left.+\overset{ˆ}{A}\left\{\left(\chi_{DRH}^{\mathrm{core}}\chi_{HOMO}^{2}\chi_{LUMO}^{\beta }\right)\left(\phi_{{SiH}_{3}}^{\mathrm{core}}\phi_{HOMO}^{\alpha }\right)\right\}\right]$$

where $C_{CS}^{20}$ and $C_{CS}^{02}$ are coefficients for the structural weights of the two doubly occupied configurations. In Equation (52), the closed-shell (CS) diradical reactant state (superscript R) is written as a linear combination of doubly occupied HOMO (HO) and LUMO (LU) of the butadiene diradical (DR) in the presence of SiH_4_, in which the doubly occupied HOMO indicates the orbital describing the reacting Si-H bond. Similarly, the open-shell (OS) reactant configuration is defined in Equation (53), but the spin coupling comes from α and β electrons in the singly occupied HOMO and LUMO orbitals of butadiene. For the product state (P) in Equation (54), in which a hydrogen atom is abstracted from SiH_3_, the spin-pairing coupling of the resulting diradical derives from two electrons in the singly-occupied orbitals (initially LUMO of butadiene and HOMO of SiH_4_) on each molecular fragment. In all, a total of six block-localized determinant configurations are included in the MAS for this biradical reaction.

**FIGURE 13 |:**
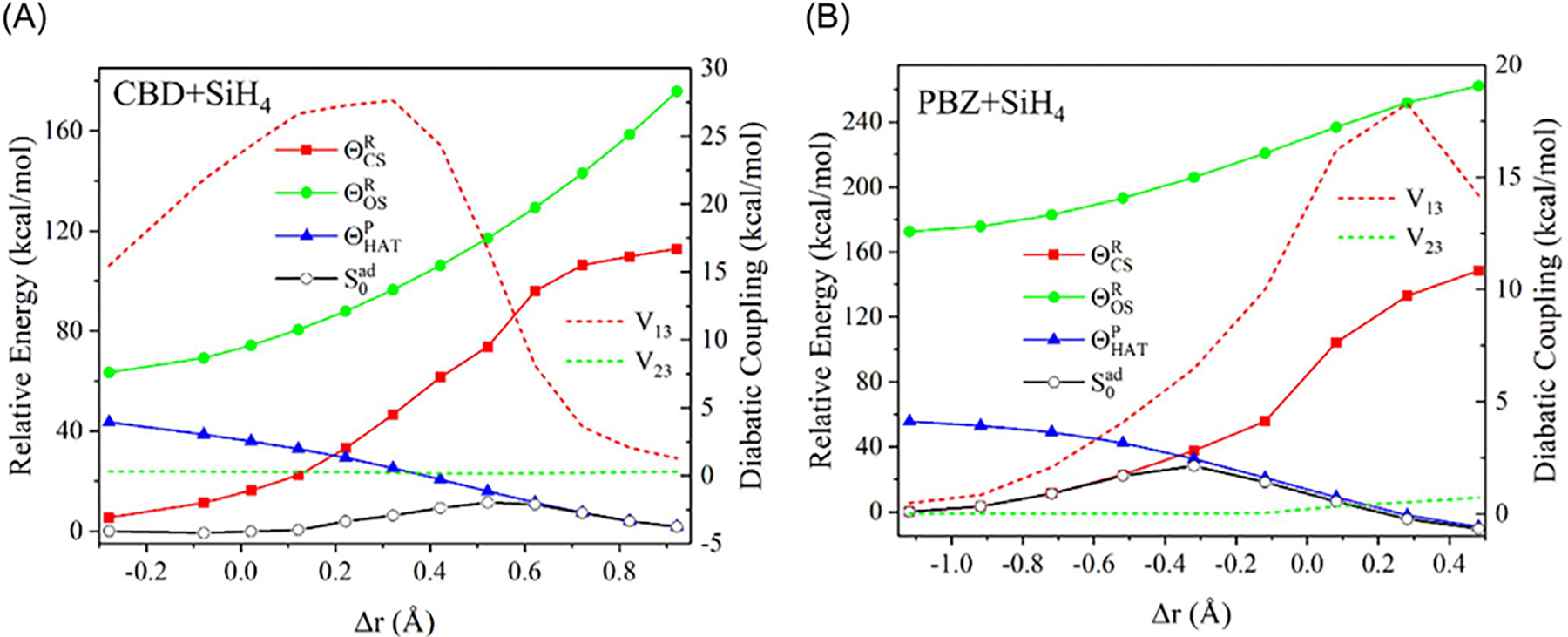
Adiabatic ground-state ($S_{0}^{\mathrm{ad}}$) and diabatic potential energy curves for the hydrogen atom abstraction of ${SiH}_{4}$ by cyclobutadiene (CBD) in (A), and by p-benzyne (PBZ) in (B). The reaction coordinate is defined as $\Delta r=r_{Si-H}-r_{C-H}$. The M06-HF functional and the cc-pVTZ basis set were used in MSDFT calculations. Adapted from [147] with permission by MDPI, Basel, Switzerland.

Figure 13 shows the potential energy curves for the singlet adiabatic ground state ($S_{0}^{\mathrm{ad}}$) and the three diabatic states defined in Equations (52–54) as well as the coupling matrix elements between the radical reactant states and the product state: ($V_{13}$ and $V_{23}$). Of notes are (1) the open-shell diradical state has the highest energies for both reactions in Figure 12, (2) there is little coupling between the OS reactant state and the product state ($V_{23}$), remaining little changed along the reaction path, and (3) interaction between the CS state with the product state is strong for both π-type and σ-type reactions, reaching a maximum in the transition state region on the adiabatic ground state. These findings shed light on the reaction mechanism taking place via the closed-s hell biradical configuration, which is in exact agreement with conclusions derived from multiconfigurational wave function approach by Hoffman and coworkers [152].

Quantitatively, the HAT barriers are determined to be 12 and 28 kcal/mol, respectively, by cyclobutadiene and by parabenzyne using MSDFT. Further, the computed energies of reaction are −2 and −1 kcal/mol from MSDFT. For comparison, the former reaction was studied by Hoffman and coworkers, who gave an energy barrier of 15 kcal/mo and reaction energy of 2 kcal/mol, using CCSD(T)/cc-pVTZ. Based on MSDFT calculations, it was further pointed out that the closed-shell mechanism formally corresponds to an electron transferred from the $\sigma_{Si-H}$ bond to the BLKS-LUMO of the biradicals. However, the origins of the CS nature in the two cases are different. For butadiene, symmetry breaking results from Jahn-Teller distortion of the molecular geometry, giving rise to a rectangular shape. On the other hand, through-bond interactions between the σ-bonds lead to significant energy separation, favoring the CS diradical character.

### 6.5 | Electron Transfer and Proton-Coupled Electron Transfer

#### 6.5.1 | Electron Transfer

The transfer integral or electronic coupling between electron (or hole) donor and acceptor groups is a key parameter for determining the rate of electron transfer and excitation-energy transfer reactions. A prerequisite for computing transfer integrals is the definition of electronically localized donor and acceptor states called diabatic states. Since diabatic states are not unique, a plethora of techniques have been developed. The block-localized Kohn–Sham determinant and BLE optimization method in MSDFT are especially suited for this purpose and have been used in a number of applications. Here, we highlight a study by Ren et al. [153] on the computation of the electronic coupling for electron-transfer reactions.

**FIGURE 14 |:**
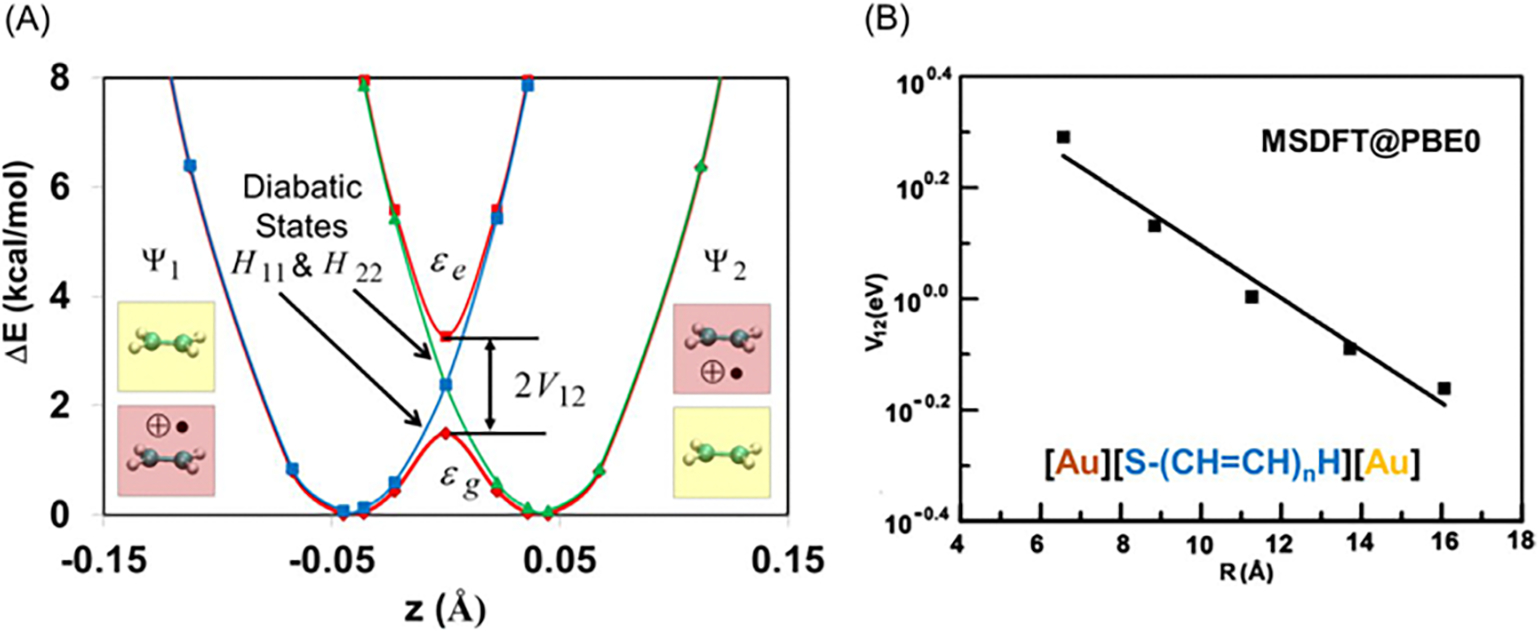
(A) Schematic illustration of the potential energy surfaces for the reactant $(H_{11}$, blue) and product $(H_{22}$, green) diabatic states and the adiabatic ground $\left(\epsilon_{g}\right)$ and excited $\left(\epsilon_{e}\right)$ states (both in red) for the electron transfer between ${CH}_{2}={CH}_{2}$ and ${CH}_{2}={CH}_{2}^{•}$. (B) Exponential attenuation of the computed transfer integral (eV) with donor-acceptor distance R for the AuS−(CH=CH)nH Au system using MSDFT with PBE0/SBKJC. Adapted from [153] with permission from the American Chemical Society.

In MSDFT, the initial ($\Psi_{1}$) and final ($\Psi_{2}$) diabatic states are defined by BLKS determinants illustrated in Figure 14A, in which one ethene molecule and one ethene cation radical are respectively partitioned into two fragmental blocks. The adiabatic ground and excited states associated with the ET process are expressed as linear combinations of the two diabatic states. The electronic coupling or transfer integral, $V_{12}$, at the transition state (the diabatic state crossing point) can be determined as half of the energy gap between the two states: $V_{12}=\left(\epsilon_{e}-\epsilon_{g}\right)/2$ from standard electronic structure calculations. In MSDFT, the transfer integral can be directly determined using the elements of the Hamiltonian matrix functional as follows [153]:

(55) $$V_{12}=\frac{1}{1\;-\;S_{12}^{2}}\left|H_{12}-\frac{H_{12}\;+\;H_{22}}{2}S_{12}\right|$$

where $S_{12}$ is the overlap integral between $\Psi_{1}$ and $\Psi_{2}$. Figure 14B plots the change of the coupling energy for a hole transfer between two Au atoms, one of which is covalently linked to an all-trans polyene and the other non-covalently at 2.5 Å away from the opposite terminal carbon atom. Conjugated π-bridges in electron transfer have been extensively studied experimentally and computationally. An important parameter in these studies is the decay rate with donor-acceptor separation; a value of $\beta =0.22\;{Å}_{-1}\left(R^{2}=0.987\right)$ was obtained, which may be compared with the experimental estimate of $0.2–0.3\;{Å}_{-1}$ [154].

**FIGURE 15 |:**
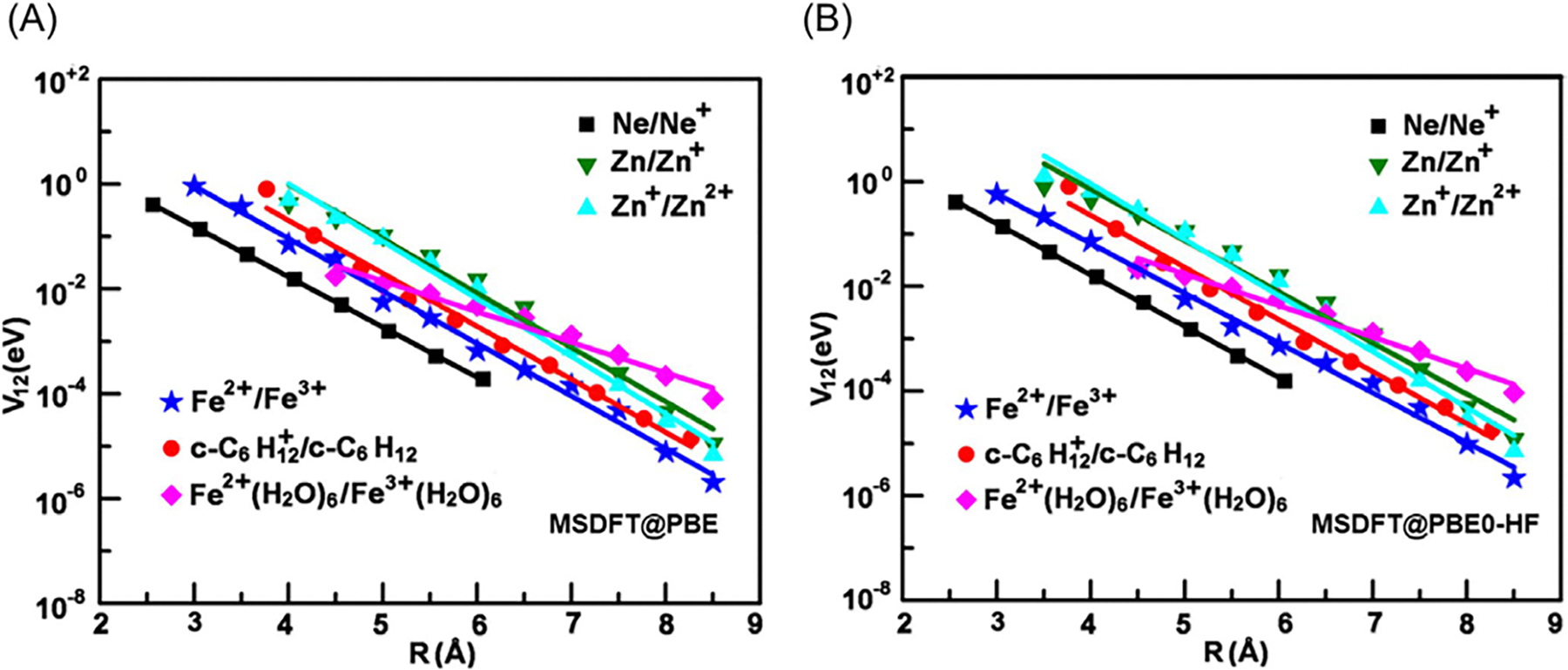
Computed transfer integral (eV) using MSDFT as a function of inter-fragment distance for self-exchange electron transfer reactions. (A) PBE/6–31+G(d) was used except for metal atoms for which the TZV basis was used. (B) PBE0 functional was used for the diagonal matrix elements and 100% Hartree–Fock exchange in the off-diagonal $H_{12}$. Adapted from [153] with permission from the American Chemical Society.

Mavros and Van Voorhis [155] found that the computed electronic coupling (transfer integral) using constrained DFT/configuration interaction (CDFT/CI) does not decay exponentially with distance and it can vary by 6 orders of magnitude simply by changing the amount of Hartree–Fock exchange. Therefore, they concluded that “it can be difficult to determine whether the CDFT-CI coupling for a particular system is trustworthy” [155]. Dexter first pointed out the exponential dependence of electronic coupling with distance since it is approximately related to the overlap $S_{12}$ between the donor $\Psi_{1}$ and acceptor $\Psi_{1}$ states [156]:

(56) $$V_{12}=V^{o}S_{12}\propto e^{-\beta R/2}\;$$

where $V^{o}$ is a constant. MSDFT was used to examine those systems encountered in reference [155], and it was found in Figure 15 that the computed $V_{12}$ values are strictly dependent exponentially with distance between donor and acceptor sites, and $V_{12}$ is also insensitive to the percentage of Hartree–Fock exchange used in a hybrid functional, visually indistinguishable in the logarithmic scale with the use of PBE functional and PBE0-HF hybrid functionals [153].

For molecular systems, the effect of exact exchange on computed electronic coupling was examined on a series of dimeric complexes in the HAB11 database [157] by using the PBE, PBE0 and PBEC functionals, which contains 0%, 25% and 100% Hartree–Fock exchange [158]. The 11 π-conjugated dimers were constructed based on size, the number of multiple bonds and varying number of heteroatoms (N, O, and S), and electronic coupling was approximated by $V_{12}^{HAB11}=0.5\left(E_{S_{1}}-E_{S_{0}}\right)$ from MRCI, CASPT2 and NEVPT2 calculations [157]. In MSDFT calculations, six structures of different inter-fragment separations for each dimer were examined, ranging from 4.5 to 7.0 Å. In that work, only two diabatic states were included in the MAS, similarly constructed as that in Figure 14A using the aug-cc-pVTZ basis functions. There was a slight dependence on the functional used in the computed electronic coupling with mean-unsigned-differences (MUD) of 23.1, 21.0, and 17.0 meV relative to the HAB11 reference data, from PBE, PBE0 and PBEC functionals, respectively. However, the difference among the three functionals is negligibly small.

Based on the computed $V_{12}$ values at various inter-monomer separations, the electronic-coupling decay rate constants, β, were extracted from their exponential dependence and are summarized in Table 5. In addition to employing Equation (55), the study also estimated the electronic coupling using $V_{12}^{\prime }(R)=\left|H_{12}(R)-\epsilon_{g}S_{12}\right|$, where R is the inter-monomer distance and $\epsilon_{g}$ is the ground state energy. Remarkably, the coupling values determined by these two methods are very similar. Moreover, Table 5 demonstrates that the trends in electronic coupling—measured by its decay rate with distance using MSDFT with a two-configuration MAS—are in excellent agreement with those obtained from high-level wave function methods that typically employ much larger active spaces.

#### 6.5.2 | Proton-Coupled Electron Transfer

**FIGURE 16 |:**
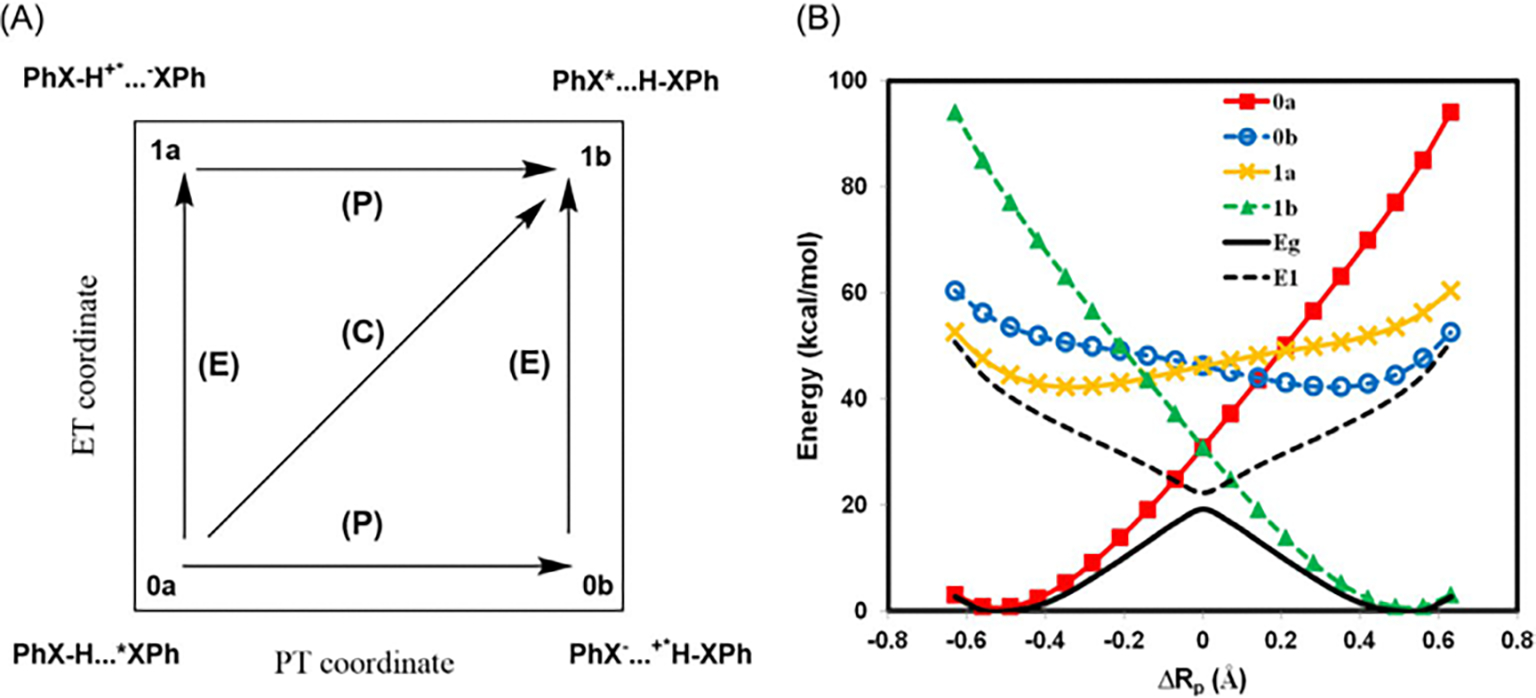
(A) Schematic representation of the mechanism for the coupled transfer of proton and electron as a function of electron transfer (E) and proton transfer (P) coordinates. (B) Computed potential energy profiles for the diabatic states corresponding to the four corners in the More O’Ferrall–Jencks diagram (A) for the hydrogen atom transfer reactions in phenol⋯phenoxyl radical. The proton coordinate, $\Delta R_{P}$, is defined as the relative distance of the transferring hydrogen from the donor and acceptor atoms. Adapted from [159] with permission by the American Chemical Society.

We also applied MSDFT to describe proton-coupled electron transfer (PCET) reactions, especially for the purpose of understanding the step-wise and concerted pathways in these processes. This is illustrated by the construction of the third dimension—electronic excited states—in a More O’Ferral–Jencks-like diagram in the formally hydrogen-atom exchange reaction of (PhX)_2_H∙, where X = O, NH, and CH_2_. Here, the four corners specifying reactant, product and single-step proton and electron transfer diabatic states can be formally defined by BLKS determinants with specific charge and spin localizations in well-defined fragmental orbital spaces. Then, the formally HAT and concerted electron-proton transfer CEPT reaction mechanisms can be written as linear combinations of these four diabatic states.

(57) $$\Omega_{R}^{HAT}=c_{0a}\Phi_{0a}+c_{1a}\Phi_{1a}$$

(58) $$\Omega_{P}^{HAT}=c_{0b}\Phi_{0b}+c_{1b}\Phi_{1b}$$

(59) $$\Omega_{R}^{CEPT}=c_{0a}^{\prime }\Phi_{0a}+c_{0b}^{\prime }\Phi_{0b}$$

(60) $$\Omega_{P}^{CEPT}=c_{1a}^{\prime }\Phi_{1a}+c_{1b}^{\prime }\Phi_{1b}$$

where $\Phi_{0a},\;\Phi_{0b},\;\Phi_{1a}$, and $\Phi_{1b}$ are the BLKS determinants representing the reactant, proton-transfer, electron-transfer and product states, respectively, in Figure 16. As one can see, each HAT state ($\Omega_{R}^{HAT}$ or $\Omega_{P}^{HAT}$) corresponds to a proton state (a and b) that is a resonance of the electronic states; in other words, electron is always in equilibrium with a given proton nuclear position. On the other hand, the proton-coupled electron transfer states ($\Omega_{R}^{\text{CEPT}}$ or $\Omega_{P}^{\text{CEPT}}$) are stabilized by the admixture of the protonic positions (0 and 1).

**FIGURE 17 |:**
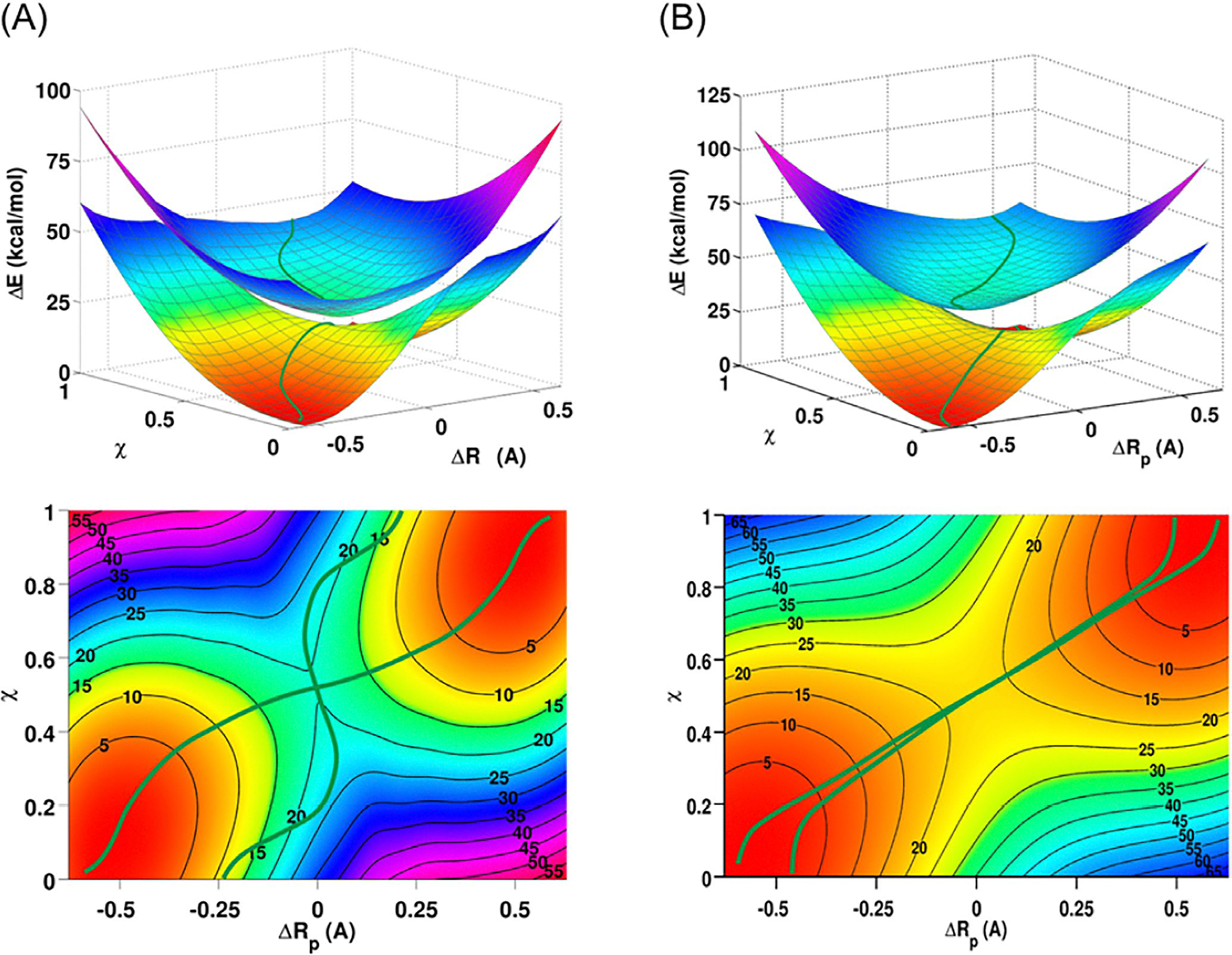
(A, top) Three-dimensional More O’Ferral–Jencks diagram for the adiabatic ground state and the first excited state as a function of the proton transfer $\left(\Delta R_{P}\right)$ and the electron transfer (χ) coordinates for the concerted **electron**-proton transfer between phenol and phenoxyl radical. (bottom). Projection of the minimum energy paths of the adiabatic ground state (green) and the excited state (red) on to the isoenergy contours for the ground state—that is, a traditional More O’Ferral–Jencks diagram. (B) 3D and 2D path-projection More O’Ferral–Jencks diagrams for the proton and electron transfer between aniline and PhNH^⋅^ radical. Multistate DFT is used in all calculations with the B3LYP functional along with the 6–31G(d) basis set. Adapted from [159] with permission from the American Chemical Society.

The three-dimensional (3D) More O’Ferral–Jencks potential energy surfaces for the phenol-phenoxy radical and aniline-anlinyl radical hydrogen exchange reactions are shown in Figure 17. The nature of the coupled electron and proton transfer in the phenol system is well-characterized to be a concerted electron-proton transfer (CEPT) process. Hammes–Schiffer has campaigned the concept of electronic non-adiabaticity being responsible for CEPT, featuring small energy gaps between the ground and the first excited states [160, 161]. Thus, the CEPT mechanism must be investigated using non-adiabatic dynamics methods. Figure 17A vividly illustrates this description showing a computed energy gap of just 3.0 kcal/mol, but, interestingly, when the minimum energy path on the $S_{1}$ excited state is projected to the 2D More O’Ferral–Jencks PES, it was found that the reaction paths on the two electronic states are nearly perpendicular to each other. This highlights the weak coupling between the two electronic states and, thus, critical role of non-adiabatic effects.

For comparison, the aniline analogue exhibits an energy gap of 20 kcal/mol between the ground and excited states, whereas that for the toluene counterpart is 128 kcal/mol (not shown)—a result that is consistent with a well-known adiabatic ground state HAT reaction. Notably, the minimum-energy projection of the excited state surface onto the ground state surface reveals parallel pathways, which increase the energy gap between the two surfaces and reinforce the adiabatic nature of the hydrogen transfer process.

#### 6.5.3 | Nuclear-Electron Orbital NEO-MSDFT

Proton tunneling is widespread in chemistry and biology [162]. One approach to meet the challenge of treating nuclear quantum effects and electronic states on the same footing is the nuclear-electron orbital (NEO) method developed by Hammes–Schiffer and coworkers [163]. The combination of NEO with MSDFT (NEO-MSDFT) t o define localized electronic states with respect to the proton transfer diabatic states is both chemically intuitive—providing insights into the understanding of proton tunneling—and quantitatively accurate by including nuclear-electron correlation at the stage of constructing diabatic states.

Yu and Hammes-Schiffer [163] first introduced a pair of localized nuclear-electronic wave functions to define the initial and final states of a proton transfer process in a double-well potential such as that in malonaldehyde and acetoaldehyde:

(61) $$\Psi_{0}=C_{A}^{0}\Phi_{A}+C_{B}^{0}\Phi_{B}$$

(62) $$\Psi_{1}=C_{A}^{1}\Phi_{A}+C_{B}^{1}\Phi_{B}$$

where $\Phi_{X}=\Phi_{X}^{e}\Phi_{X}^{p}$ is the electronic $\Phi_{X}^{e}$ and protonic $\Phi_{X}^{p}$ wave functions localized at site X where X=A,B, and {C} are the configuration coefficients for the ground and excited states of the migrating proton. The NEO-MSDFT states in Equations (61) and (62) are obtained by solving the generalized secular equation of NOSI, and the solution includes coupled p−p,e−e, and e−p interactions. In the NEO-MSDFT approach, the nuclear-orbital coupling term between two diabatic states is determined by [163].

(63) $$H_{AB}=\left\langle \Phi_{A}\right|{\overset{ˆ}{H}}_{NEO}\left|\Phi_{B}\right\rangle +\frac{\lambda }{2}\left(E_{A}^{\text{corr}}+E_{B}^{\text{corr}}\right)$$

where ${\hat{H}}_{\text{NEO}}$ is the NEO Hamiltonian and the correlation energies for the nuclear-orbital diabatic states are determined by NEO-BLKS DFT and HF calculations: $E_{X}^{\text{corr}}=E_{X}^{NEO-KS}\left[\rho_{X}^{e},\rho_{X}^{p}\right]-E_{X}^{NEO-HF}\left(\Phi_{X}\right)$.

The NEO-MSDFT method has been extended to the simultaneous transfer of any number of M protons [164, 165]. In this case, a total of $2^{M}$ block-localized states will be first optimized to account for all possible combinations of proton tunneling process, leading to a $2^{M}x2^{M}$-dimension NOSI-Hamiltonian that is diagonalized to obtained the NEO wave functions and energies. Recently, analytic gradients for the NEO-MSDFT energy functional have been implemented to carry out both adiabatic and non-adiabatic dynamics simulations [164, 165].

**FIGURE 18 |:**
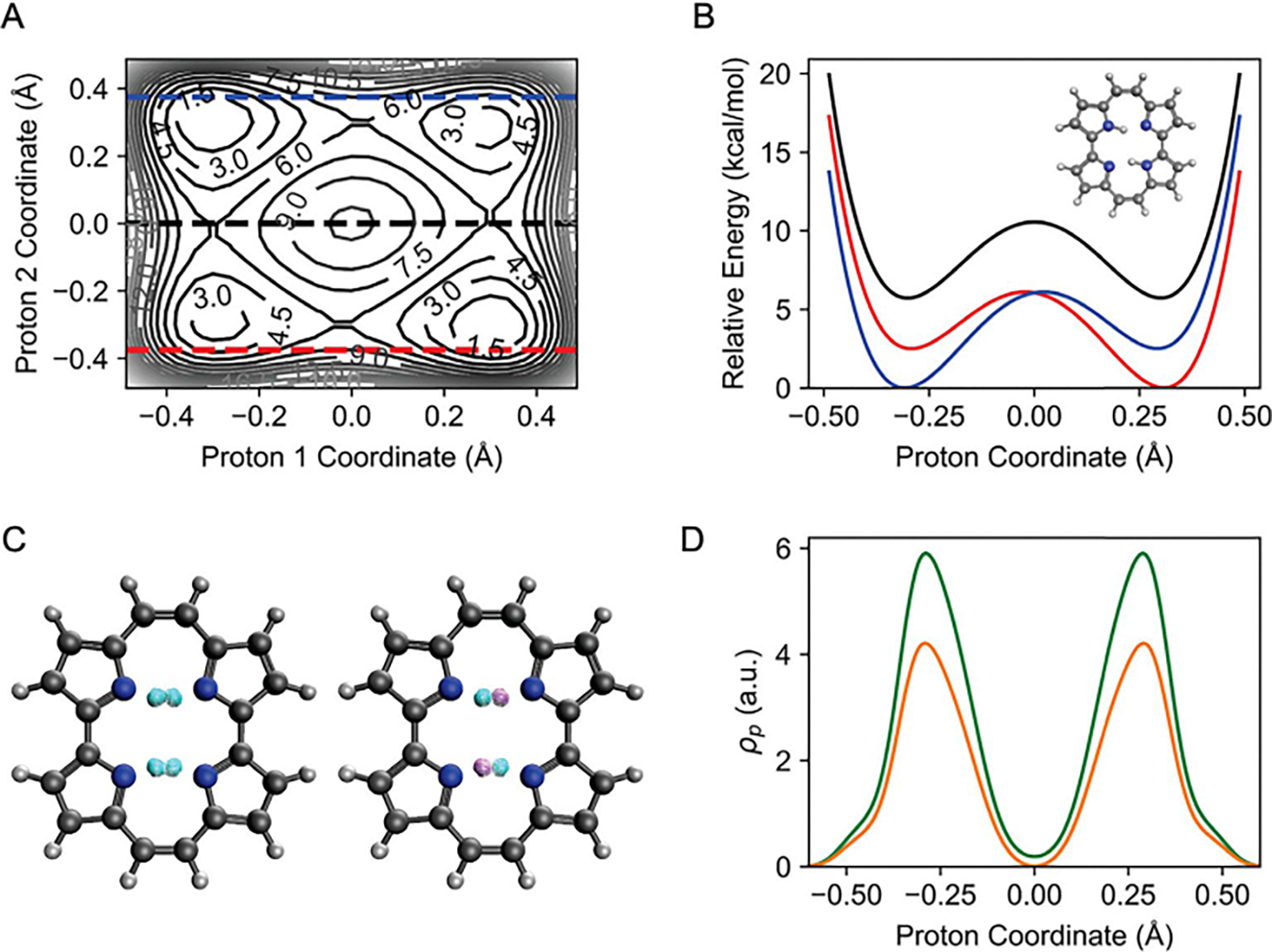
(A) Two-dimensional proton potential energy surface as a function of the two proton coordinates, each corresponding to a one dimensional grid spanning the line connecting the proton positions optimized at the conventional DFT level for the two trans structures with the origin at the midpoint between them. Relative energies in kilocalories per mole are provided on each contour line. (B) One-dimensional proton potential energy curves corresponding to slices of the two-dimensional surface along the proton 1 coordinate with the proton 2 coordinate fixed to positions of −0.375 Å (red), 0 Å (black), and 0.375 Å (blue). The corresponding colored dashed lines of panel A indicate these one-dimensional slices on the two-dimensional potential energy surface. (C) Ground state (left) and first excited state (right) NEO-MSDFT proton densities. To visualize the phase of the corresponding proton vibrational wave function, the positive and negative phases are colored cyan and purple, respectively. (D) Ground state (green) and first excited state (orange) proton densities computed with NEO-MSDFT along one of the proton transfer coordinates. Adapted from [166] with permission by the American Chemical Society.

A range of one and two proton transfer reactions have been studied by using the NEO-MSDFT method. We highlight a double proton tunneling in porphycene studied by Dickinson et al. [166]. Shown in Figure 18 are the two-dimensional potential energy surface and one-dimensional slices, both in the protonic ground and excited states, along the two proton coordinates. A number of notable features were found by these authors, including a symmetry double well potential slice from the coupling of asymmetric PEC for each proton-electron diabatic state, symmetric NEO wave function in the ground state but antisymmetric in the first excited state, and a finite proton density (probability) at the transition state (proton coordinate value at 0) on the ground state, but a node of zero probability at that coordinate on the excited state. The computed tunneling splitting is 44.84 cm^−1^, which is in excellent agreement with the 2D FGH (Fourier grid Hamiltonian, a numerically exact) method at 37.63 cm^−1^ [166].

### 6.6 | Light-Harvesting Complex II

**FIGURE 19 |:**
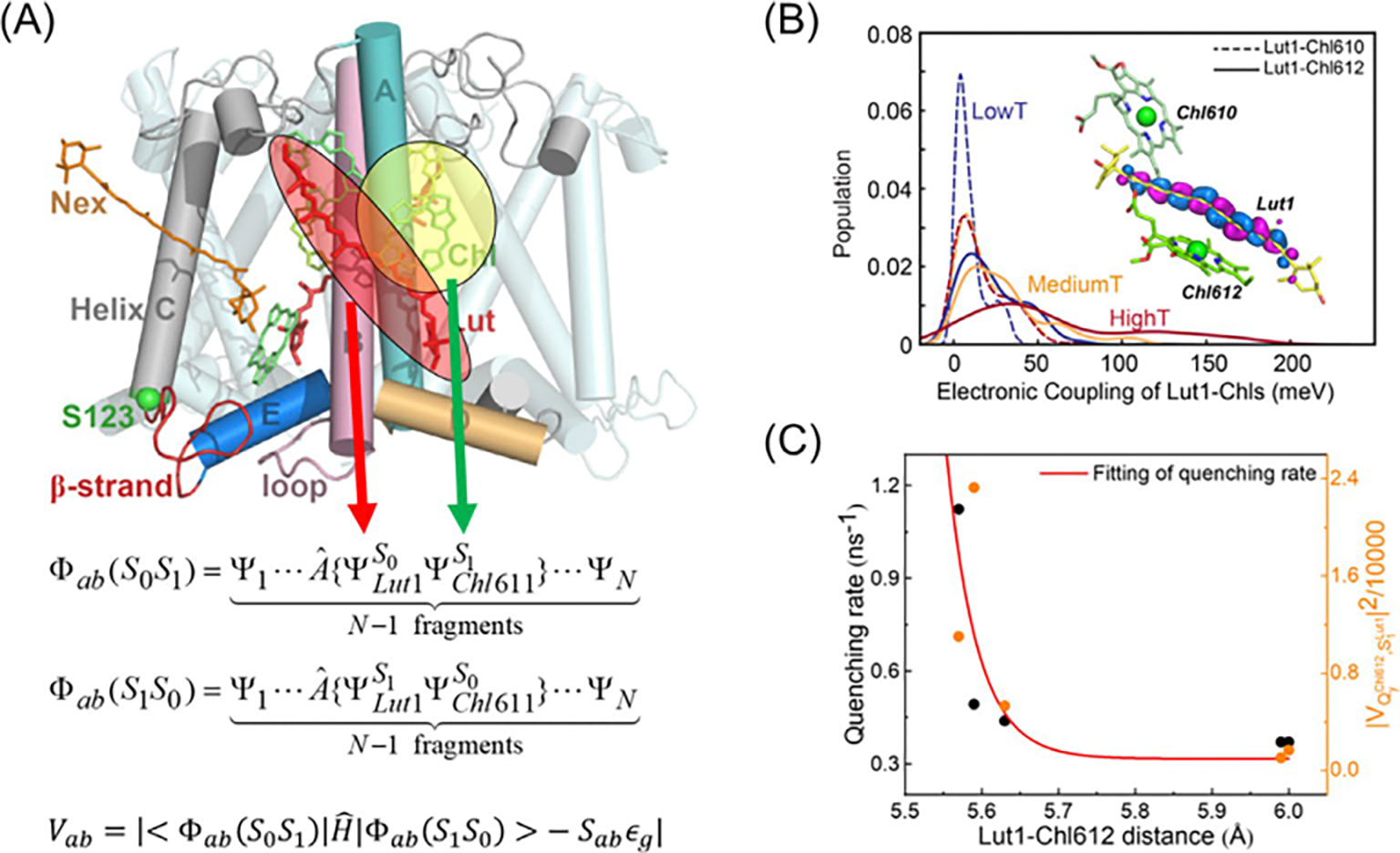
(A) Schematic illustration of light-harvesting complex II and the initial excited state chlorophyll 612 (Chl612), $\Phi_{ab}\left(S_{0}S_{1}\right)$ and the final $S_{1}$ state of Lutein 1 (Lut1), $\Phi_{ab}\left(S_{1}S_{0}\right)$. Electronic coupling $V_{ab}$ between the two states are computed using MSDFT. (B) Distribution of temperature-induced electronic couplings between Lut1 and Chl610 and Chl612 in one monomer of LHCII. The computed electronic couplings are given as dashed curves for Lut1-Ch1610, and as solid curves for Lut1-Ch612 at low (270−290K) in blue, medium (300−320K) in orange, and high (330−360K) in maroon temperature ranges. The CAM-B3LYP functional and 6−31G(d) basis set was used in MSDFT calculation of the coupled chromophores embedded in a molecular mechanical (QM/MM) environment of the protein and solvent. (C) The relationship between fluorescence decay rate k=1/(fluorescence lifetime) (black circles), Lut1-Chl612 electronic coupling strength ${\left|V_{Qy}^{\text{Ch1612-Lut1}}\right|}^{2}/10,000$ (orange circles) against Lut1-Chl612 distance (R) and the fitting equation is $k=0.31+0.31x10^{-25(R-5.6)}$ (the fitted line is in maroon). Adapted from [167, 168] with permission by Springer Nature.

Excited-state energy transfer processes are widespread from biological photo-systems and photo-receptors to artificial photovoltaic materials. Block-localization of electronic excited states with specific charge and spin is ideally suited for these applications, and MSDFT-NOSI has been used to characterize the mechanism of singlet fission [126] and non-photochemical quenching (NPQ) in the major light-harvesting complex of photosystems II (LHCII) among other problems of chemical and biological interest (Figure 19) [167, 168].

We conclude our sampling of applications of MSDFT with a biophysical application relevant to photosynthesis. In photosystem II, the antenna protein light-harvesting complex (LHCII) in higher plants regulates the reversible switch between an efficient light-harvesting process and a photoprotection state called non-photochemical quenching. The fast energy dissipation step takes place in the LHCII complex, now recognized between Chlr612 and Lut1. Although this decade-old, multiscale problem has been well-understood at the cellular level, a mechanism in the atomic detail was not available until 2020 [167]. Through a set of molecular dynamics simulations of LHCII in a membrane environment at different temperatures, coupled with MSDFT calculations (Figure 19A), it was discovered that an allosteric protein conformational change of two trans-membrane α-helices (per monomer of the trimeric LHCII) leads to enhanced electronic coupling between the bright $S_{1}$ state of Chlr612 and the dark $S_{1}$ state of Lut1 at high temperatures to mimic the full sunshine condition (Figure 19B), thereby, increased quenching rate for energy dissipation [167].

The predicted structural change and enhanced quenching rate as a result of protein allosteric regulation was reproduced 3 years later through cryo-EM structural determination under high and low acidity conditions and in detergent and nanodisc environment [168]. The predicted local secondary structural transitions from 3 to 10 E-helix and C-terminal coil to two α-helices that induce conformational change of trans-membrane helices were confirmed by these experiments [168]. Furthermore, it was found that the experimental quenching rates under different pH conditions and aggregation levels correlate with the computed electronic coupling values [168]. MSDFT computational results along with the corresponding experiments conclude that LHCII is a molecular machine, acting as a quantum switch to rapidly change between the light-harvesting quantum state and the energy dissipating quenched state (Figure 19C).

## 7 | Concluding Summary

MSDFT extends the theorems of Hohenberg and Kohn to encompass both ground and excited electronic states. By introducing a matrix density D(r) of rank N as the fundamental variable, MSDFT establishes a one-to-one correspondence between D(r) and the Hamiltonian matrix associated with these N electronic states—that is, a matrix density functional ℋ[D]. Notably, this N-matrix density can be exactly represented by no more than $N^{2}$ Slater determinants. Establishing an upper bound for density representation gives rise to a new concept in treating excited states—the MAS. As a result, the computational efficiency inherent to ground-state Kohn–Sham DFT is effectively extended to excited-state calculations.

This review introduces the fundamental theory and methods of MSDFT. It covers the existence of a universal matrix functional defined on the Hilbert subspace spanned by the lowest N eigenstates, the multistate variational principle employed to optimize the N-matrix density and determine the energies and densities of individual eigenstates, and the definition of a correlation matrix functional with respect to the MAS. Moreover, due to the inherent symmetry of the Hilbert space, the structure of electron correlation in the N-dimensional subspace within MSDFT is strictly ordered; all elements of the correlation matrix functional are interrelated. In fact, knowing any one diagonal element—a single state correlation functional $D_{AA}[D]$, 1≤A≤N—uniquely determines the remaining $\left(N^{2}-1\right)$ elements. These fundamental theorems not only provide deeper insights into electron correlation but also open avenues for developing novel approximations for the correlation matrix functional that treat all electronic states equally. Clearly, this represents a major goal for the near future as it will extend the accuracy of computation.

The concept of a MAS opens up the possibility of developing a variety of computational methods and adopting techniques that have been well established in wave function theory, including configuration interaction and multiconfiguration self-consistent-field methods. In this review, we focus on the method and application of NOSI—a wave function-based non-orthogonal configuration interaction analog that incorporates correlation effects. The examples highlighted in this article range from excitation energies of closed-shell and open-shell valence, core-level, and charge-transfer excitations to processes such as chemical reactions, electron transfer, proton-coupled electron transfer, and excited-state energy transfer. Echoing the sentiment expressed in the introduction, we hope that the theory, methods, and applications presented here could stimulate the development of even more sophisticated computational approaches, thereby further expanding the scope of applications.

## Figures and Tables

**TABLE 1 |:** Energies for the ground state ($S_{0}$) and triplet state ($T_{1}$) computed using MSDFT and KS-DFT.

Molecule	$S_{0}$		$T_{1}$	
	MSDFT	$\Delta E_{\mathrm{KS}}$	MSDFT	$\Delta E_{\mathrm{KS}}$

Loos BTEDB1				
H_2_O	−76.43070	−0.00048	−76.15937	0.00000
H_2_Sb	−399.39358	−0.00019	−399.18412	−0.00239
NH_3_	−56.55466	−0.00148	−56.31586	−0.00001
HCl	−460.80965	0.00000	−460.53647	0.00000
HC≡CH	−77.32602	0.00000	−77.11100	0.00000
H_2_C=CH_2_	−78.57326	0.00000	−78.40807	0.00000
Cyclopropane	−116.61033	0.00000	−116.45067	0.00000
H_2_C=O	−114.50102	−0.00169	−114.37185	0.00000
CH_3_CH=O	−153.78187	−0.00063	−153.63980	−0.00006
CH_2_=C=O	−152.56190	0.00000	−152.42677	0.00000
HCONH_2_	−169.85401	−0.00052	−169.65987	−0.00003
H_2_C=S	−437.45851	0.00000	−437.39160	0.00000
CH_2_=NH	−94.62199	−0.00012	−94.45830	−0.00001
CH_3_–N=O	−169.79038	−0.00054	−169.75579	−0.00009
H_2_C=N_2_	−148.73554	−0.00044	−148.63877	0.00020
Streptocyanine	−150.37478	0.00000	−150.17170	0.00000
CO	−113.32105	−0.00000	−113.09571	0.00000
N_2_	−109.53515	−0.00002	−109.25536	−0.01503
MS-EDA Monomers				
Naphthalene	−385.76533	0.00000	−385.63259	−0.00050
Pentacene	−846.54223	0.00000	−846.50452	−0.01088
PCA	−845.54485	−0.00007	−845.40970	−0.03489
TCNE	−447.41678	0.00000	−447.31172	0.00000
t-DME	−307.51100	−0.00015	−307.35069	−0.00004
c-DME	−307.50997	−0.00028	−307.35079	−0.00026
MVE	−193.03249	−0.00043	−192.86691	0.00000
Me_2_C=O	−193.07551	−0.00055	−192.92376	0.00000
Me_2_C=O (S1)	−193.04394	−0.00197	−192.94603	−0.00001
Pentacene (S1)	−846.53459	0.00000	−846.51462	−0.00923

**TABLE 2 |:** Statistics in mean signed error (MSE), mean absolute error (MAE), root-mean-square error (RMSE), and Maximum Deviations of Computed Excitation Energies (in eV) with respect to the theoretical best estimates obtained by using MSDFT-NOSI and other methods reported in reference [122].

Method	# States	MSE	MAE	RMSE	Max (+)	Max (–)

CC3	106	−0.01	0.03	0.04	−0.09	0.19
CC2	106	0.03	0.22	0.28	−0.71	0.63
CCSDT	104	−0.01	0.03	0.03	−0.10	0.11
CCSD	106	0.05	0.08	0.11	−0.17	0.40
ADC(3)	106	−0.15	0.23	0.28	−0.79	0.39
TDDFT	100	−0.24	0.31	0.42	−1.50	1.70
SMD-BLE	100	−0.02	0.17	0.22	−0.71	0.59
SMD-TSO	100	−0.01	0.18	0.24	−0.74	0.69

*Note:* The performance of the present multistate density functional theory (MSDFT) is compared with time-dependent density functional theory (TDDFT) using the same M06-2X functional [124] along with wave function approaches including coupled cluster methods (CCSD, CCSDT), the perturbative second- and third-order coupled cluster theory (CC2, CC3) and the algebraic diagrammatic construction of third order (ADC(3)).

**TABLE 3 |:** Computed first and second excitation energies (eV) of aryl-tetracyanoethene complexes in the gas phase using LR-TDDFT with different functionals and MSDFT@M06-2X along with experimental data. Reproduced from Reference [93] with permission by NPJ publisher.

Aryl-TCNE	M06-2X	BNL	BNL (γ_opt_)	CAM-QTP-02	MSDFT@M06-2X	Expt

Benzene	2.99, 3.04	4.4	3.8	3.85	3.66, 3.74	3.59 (3.67)
Toluene	2.74, 2.94	4.0	3.4	3.50	3.28, 3.63	3.36 (3.35)
o-Xylene	2.48, 2.78	3.7	3.0	3.25	3.07, 3.32	3.15 (3.15)
Naphthalene	1.94, 2.73	3.3	2.7	2.80, 3.57	2.69, 3.43	2.60, 3.23
Anthracene	1.89, 2.94	2.6	2.1	—	1.62, 2.90	3.73, 1.73, 2.79

**TABLE 4 |:** Computed and experimental core excitation energies (eV) for benzene cation in the elongated B3g minimum geometry optimized by using M06-2X/cc-pVQZ.^a^

Transition	EOM-CCSD	X-TDA	MSDFT^b^	Expt^c^

C(1s)→SOMO (E)	281.6	278.8	281.1	281.3
$\sigma_{5}\to \pi_{5}^{*}$	(285.2)	282.5	283.1	(283.5)
$\sigma_{4}\to \pi_{4}^{*}(F)$	285.8	283.0	284.1	(284.7)
$\sigma_{5}\to \pi_{5}^{*}(F)$	286.4	283.9	284.5	284.9
$\sigma_{4}\to \pi_{4}^{*}$	—	—^d^	285.3	—
$\sigma_{4}\to \pi_{4}^{*}(G)$	287.4	286.6	285.9	285.9
$\sigma_{5}\to \pi_{5}^{*}$	—	—^d^	286.0	—
$\sigma_{4}\to \pi_{4}^{*}$	288.4	—^d^	287.6	(287.6)
$\sigma_{5}\to \pi_{5}^{*}$	289.0	—^d^	288.1	(288.3)
$\sigma_{1}\to \sigma_{\text{Ryd}\;}^{*}$	—	287.8	288.7	(288.7)
$\sigma_{1}\to \sigma_{\text{Ryd}}^{*}(H)$	290.7	288.4	290.4	290.0

*Note:* Adapted from [117] with permission by the American Chemical Society.

a MSDFT and X-TDA results are obtained with the BLYP (BH& HLYP) functional and aug-cc-pVTZ basis set. EOM-CCSD data are taken from reference [136].

b Minimal active space includes configurations of the spectator radical both in the $\pi_{2}$ and $\pi_{3}$ orbitals.

c In parentheses are peaks in the experimental spectra, not specifically assigned.

d These excitations cannot be unambiguously assigned.

**TABLE 5 |:** Computed decay rate constants (Å^−1^) of electronic coupling versus inter-fragment distance for of dimer complexes in the HAB11 database using MSDFT with the PBEC functional and aug-cc-pVTZ basis set.

Dimer	$V_{12}$	$V_{12}^{\prime }$	HAB12 dataset

Ethylene	2.66	2.62	2.84
Acetylene	2.85	2.84	3.08
Cyclopropene	2.89	2.82	3.06
Cyclobutadiene	2.51	2.44	2.63
Cyclopentadiene	2.65	2.64	2.79
Furane	2.73	2.72	2.82
Pyrrole	2.62	2.59	2.79
Thiophene	2.81	2.80	2.95
Imidazole	2.67	2.66	2.72
Benzene	2.74	2.71	3.02
Phenol	2.82	2.80	3.03
MUD (Å^−1^)	0.16	0.19	
$V_{12}\mathrm{MUD}\;(\mathrm{meV})$	17.0	15.2	

*Note:* $V_{12}^{\prime }$ was estimated using $V_{12}^{\prime }=\left|H_{12}-\epsilon_{g}S_{12}\right|$. Reference values in the HAB11 dataset were determined using MRCI, CASPT2, and NEVPT2 methods.

## Data Availability

Data sharing are not applicable to this article as no new data were created or analyzed in this study.
